# Challenges in simulating and modeling the airborne virus transmission: A state-of-the-art review

**DOI:** 10.1063/5.0061469

**Published:** 2021-10-27

**Authors:** Farzad Pourfattah, Lian-Ping Wang, Weiwei Deng, Yong-Feng Ma, Liangquan Hu, Bo Yang

**Affiliations:** 1 Guangdong Provincial Key Laboratory of Turbulence Research and Applications, Center for Complex Flows and Soft Matter Research and Department of Mechanics and Aerospace Engineering, Southern University of Science and Technology, Shenzhen 518055, People's Republic of China; 2 Guangdong-Hong Kong-Macao Joint Laboratory for Data-Driven Fluid Mechanics and Engineering Applications, Southern University of Science and Technology, Shenzhen 518055, People's Republic of China

## Abstract

Recently, the COVID-19 virus pandemic has led to many studies on the airborne transmission of expiratory droplets. While limited experiments and on-site measurements offer qualitative indication of potential virus spread rates and the level of transmission risk, the quantitative understanding and mechanistic insights also indispensably come from careful theoretical modeling and numerical simulation efforts around which a surge of research papers has emerged. However, due to the highly interdisciplinary nature of the topic, numerical simulations of the airborne spread of expiratory droplets face serious challenges. It is essential to examine the assumptions and simplifications made in the existing modeling and simulations, which will be reviewed carefully here to better advance the fidelity of numerical results when compared to the reality. So far, existing review papers have focused on discussing the simulation results without questioning or comparing the model assumptions. This review paper focuses instead on the details of the model simplifications used in the numerical methods and how to properly incorporate important processes associated with respiratory droplet transmission. Specifically, the critical issues reviewed here include modeling of the respiratory droplet evaporation, droplet size distribution, and time-dependent velocity profile of air exhaled from coughing and sneezing. According to the literature review, another problem in numerical simulations is that the virus decay rate and suspended viable viral dose are often not incorporated; therefore here, empirical relationships for the bioactivity of coronavirus are presented. It is hoped that this paper can assist researchers to significantly improve their model fidelity when simulating respiratory droplet transmission.

## I. INTRODUCTION

As of August 2021, the COVID-19 virus pandemic has infected more than 216 484 958 people and caused more than 4 503 000 deaths worldwide.^1^ Several countries have been hit by multiple waves of the pandemic with mutations of the virus exhibiting stronger infectious rates. The COVID-19 virus is likely to co-exist with the mankind for a prolonged period of time. It forces researchers and health professionals to seek solutions to control and contain the spread. Although we had other pandemics of the similar kind in the past, little is known on how to quantitatively assess the physical transport and lifecycle of infectious viruses when they are airborne. Currently, the World Health Organization and government agencies had acknowledged that the COVID-19 virus could be transmitted by the airborne aerosol route.^2^

**FIG. 1.**
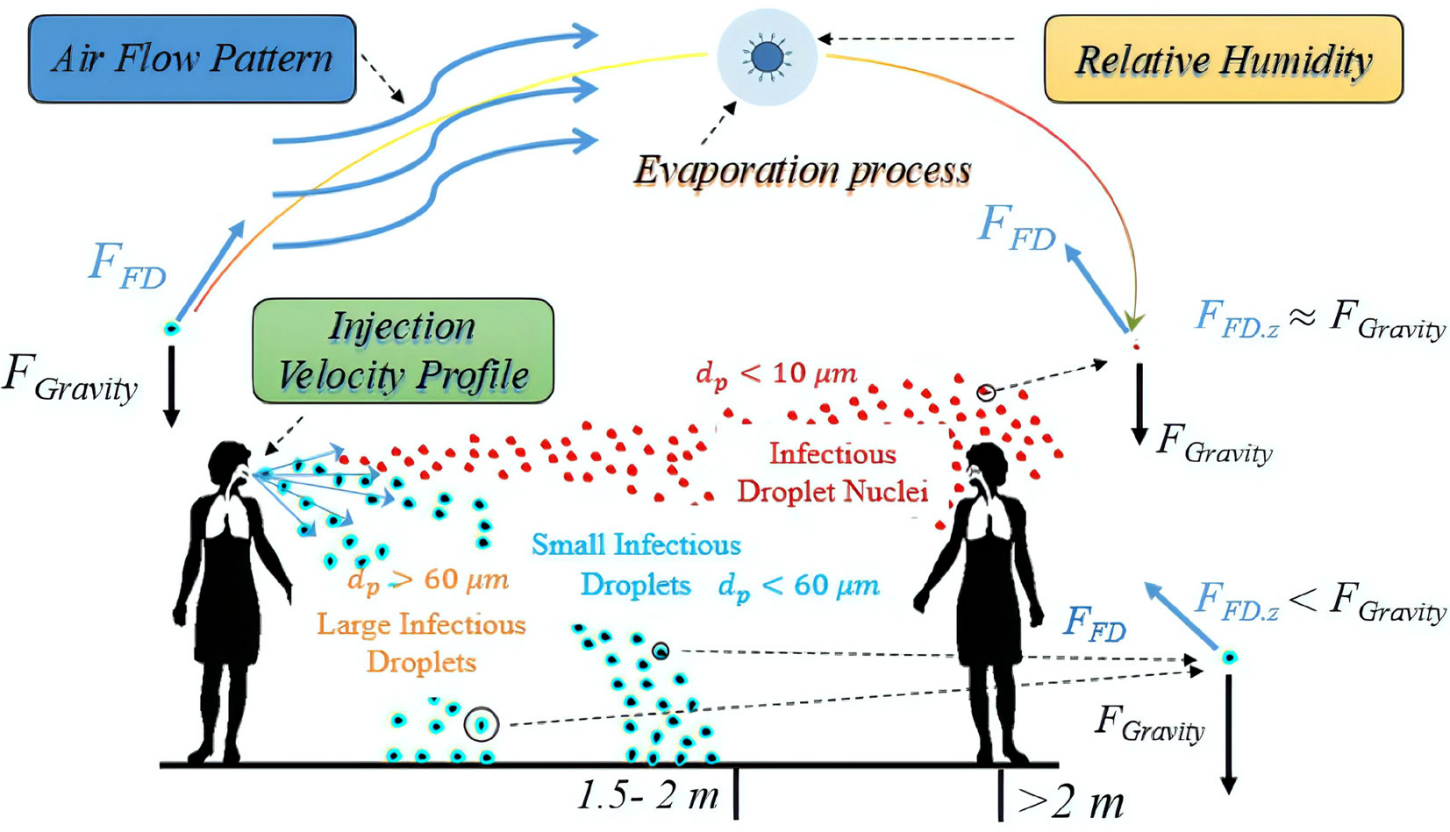
A schematic of the fate of droplets produced by human respiratory activity. Here, *d_p_* is the particle diameter, *F*_FD_ denotes the air drag force acting on the particle, *F*_Gravity_ is the gravitational body force, and the subscript *z* indicates the vertical component.

Sneezing, coughing, talking, and even the infected patient exhaling can spread the virus in the environment. These activities first generate virus-containing droplets of different sizes. These droplets are transported by their initial velocity at the mouth/nose release point and the environmental airflow, and they may settle to the ground or other surfaces by gravity. Depending on the environmental temperature and humidity, their sizes also change due to evaporation. In this review paper, the basic terminology and size ranges are given in Table I, following the literature.^3–5^ A schematic of the fate of droplets produced by human respiratory activity and the effect of particle size on their behavior is shown in Fig. 1. As can be seen, the large droplets with diameter larger than 60 μm settle to the ground typically within 10 s due to the gravity of Earth, and they are involved in the short-range transmission before evaporating.^6^ Small droplets with diameter less than 60 μm could move upward, be suspended in the air.^7^ They can be transported by the air over long distances due to the environmental airflow. The main fraction of the droplets released by coughing or sneezing is water, and water can evaporate into the air.^6^ After the water evaporates, the droplet nuclei containing NaCl, protein, and mucus remain, and they are typically less than 10 μm in diameter. The droplet nuclei could still be a significant cause of infection in the environment, as first reported by Wells in 1934.^8^ The virus-containing droplet nuclei may remain both airborne and infectious for hours.^9^ Virus-containing small droplets and droplet nuclei can be viewed as part of aerosol particles due to their similar size ranges. They may be moved by indoor and outdoor airflows and could be directly inhaled by healthy entities imposing potential infection risk. For this reason, we sometime use droplets, aerosols, and particles interchangeably.

**TABLE I.** The definition and size range of virus-containing droplets and particles.

Droplet type	Definition	Size range
Droplet nuclei	Formed from the evaporation of respiratory droplets	dp<10 μm
Small droplets	Formed from the respiratory activities	$d_{p}<60\;\mu m$
Large droplets		$d_{p}>60\;\mu m$
Aerosol particles	A suspension of solid or liquid particles in a gas	0.002 μm<dp<100 μm

Therefore, virus infection can either be transmitted by larger droplets upon direct contact over short (i.e., 2 meter-scale) social distance or by smaller airborne droplets and droplet nuclei in the air over a much longer distance range (∼10 m or more).^10^ The mechanisms for long-range and short-range transmissions are different.^11^ Disease transmission is a complex and interdisciplinary process involving microbiology, environmental science, and social science.^10^ The mechanisms of transmission of droplets and aerosols in the indoor and outdoor spaces are poorly understood,^12^ because their dispersion range depends on several factors such as the evaporation rate, flow pattern, initial particle size distribution (PSD) at the mouth/nose release point, the time-dependent velocity profile of exhaled jet during coughing and sneezing, thermal plumes associated with the warm body surface temperature,^13–15^ and environmental relative humidity and temperature.^16^ All these complexities present significant challenges when one attempts to perform numerical simulations of expiratory droplets and aerosols.

Another complexity for numerical modeling is the proper boundary conditions. The number of research that examined the dynamic flow characteristics and PSD of coughing and sneezing is considerable.^17,18^ Still, most of them are inadequate in providing the proper boundary conditions needed for the numerical simulations. The needed boundary conditions include the injection duration, time-dependent velocity profile of respiratory activity, and plume direction. Computational fluid dynamics (CFD) simulations need accurate boundary conditions, whereas no comprehensive literature exists.^19^

Finally, there are significant inconsistencies in the literature regarding the droplet sizes expelled during coughing, sneezing, breathing, and talking.^20,21^ In particular, the reported PSDs due to human respiratory activities contain apparent discrepancies. The range of particle size varies from 1 to 1000 *μ*m, and different values have been reported regarding the maximum speed of coughing and sneezing.^22,23^

**FIG. 2.**
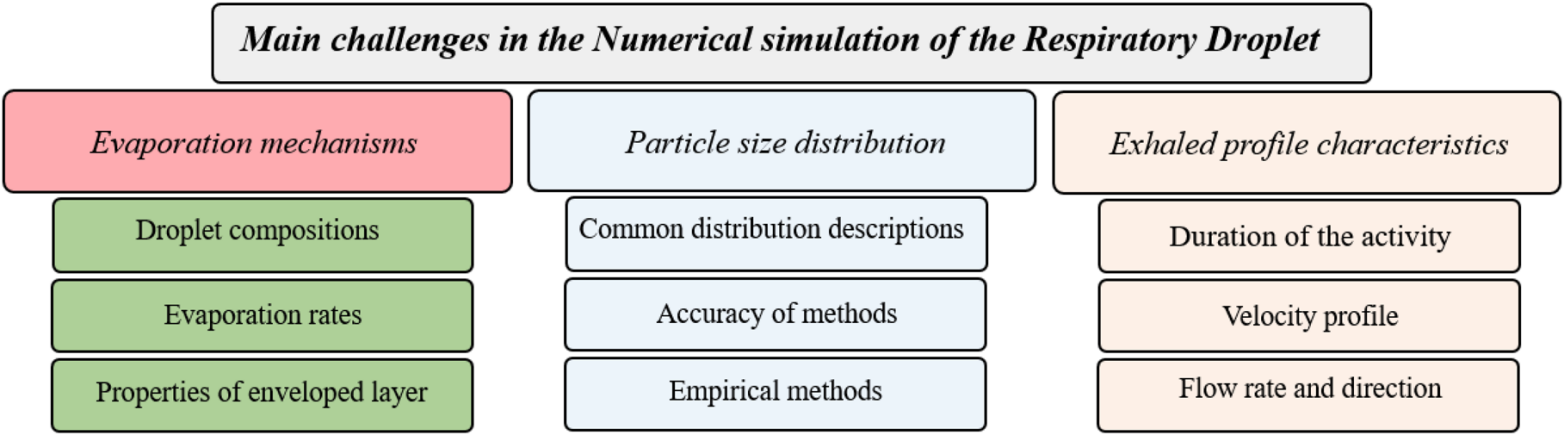
The main challenges in the numerical simulation of the transport of respiratory droplets.

Therefore, based on our understanding of the literature and our CFD knowledge and experience, main challenges in the numerical simulation of virus transport may be summarized in Fig. 2, including PSD and its mathematical description during respiratory activities, respiratory jet boundary conditions, and modeling of evaporation. The key open questions appear to include: what is the PSD from human respiratory activities; what is the time-dependent velocity profile of the expired plume (cough cloud profile); what are the evaporation rate and the changing size distribution of droplets over time; and how does the droplet chemical composition affect the vaporization rate?

This review paper aims one to prepare a guideline for more accurately modeling of human expiratory activities such as breathing, coughing, speaking, and sneezing. The presentation of the details of the state-of-the-art literature can help formulate a more realistic simulation of disease transmission through indoor and outdoor environments. The application of more realistic boundary conditions is one of the essential steps in numerical simulations. In addition, careful evaporation rate modeling is a vital step in the numerical simulation of virus transmission by droplets.

More than 300 articles were studied in preparing this review article with the keywords of numerical simulation of respiratory droplet dispersion, respiratory droplet size distribution, decay rate of the virus, and multi-component droplet evaporation modeling. These articles were categorized based on the title and abstract of the articles. A subset of about 100 articles was selected to address important issues related to the accuracy and modeling fidelity of the numerical simulation. This process led to the selection of three main issues indicated in Fig. 2.

A few recent review papers relevant to virus airborne transmission, but with different focuses, should be noted. Some pervious review papers discussed the effect of air flow pattern^24^ and ventilation arrangement^13^ on airborne transmission, as well as the measurement methods of the respiratory droplets.^17,18^ Chow and Yang^24^ reviewed numerical modeling efforts in the indoor environment, focusing on the effects of the air inlet/outlet location on airflow patterns and ventilation systems performance. They emphasized the importance of using CFD tools in the establishment of engineering standards. Li *et al.*^13^ conducted a multidisciplinary systematic review of studies published between 1960 and 2005 on the role of ventilation rates on infection transmission. Gralton *et al.*^17^ and Merghani *et al.*^18^ reviewed the literature on measuring the properties of airborne particulate matter. They pointed out that the type of the particle measurement method significantly affects the experimental results. Also, they mentioned that measuring the size distribution of mucus particles requires further research, because the size of droplets and mucus composition from infected individuals were different from healthy individuals. Also, Ai and Melikov^25^ reviewed the shape of the mouth and nose and exhaled flow characteristics. They concluded that fast measurements are required to provide accurate concentration profiles for time-dependent evaluations, especially for short-term events.

The remainder of the current review paper is organized as follows. In Sec. II, a simple model for the transmission distance of the respiratory droplet is presented to illustrate key factors affecting the travel distance of droplets. The description and modeling of PSD relevant to virus-containing droplets are discussed in Sec. III. The relevant details of boundary conditions are explained in Sec. IV. The complex issues of evaporation modeling for biologically active virus-containing droplets are reviewed in Secs. V and VI. Finally, Sec. VII concludes the paper with a summary and perspectives.

## II. A SIMPLE MODEL OF TRANSMISSION DISTANCE OF RESPIRATORY DROPLETS

As mentioned in Section I, the droplet's size has a significant effect on their fate. Larger droplets are affected by gravity and settle to the ground after a short distance, while small droplets remain suspended in the air. A rough estimation of the distance traveled by the droplets released from the respiratory activity can help establish health protocols, determine the exact social distance, and control the epidemic. In this section, a discussion about the distance traveled by droplets under different but uniform background airflow velocities is presented to briefly illustrate the importance of background air velocity and particle size.

**FIG. 3.**
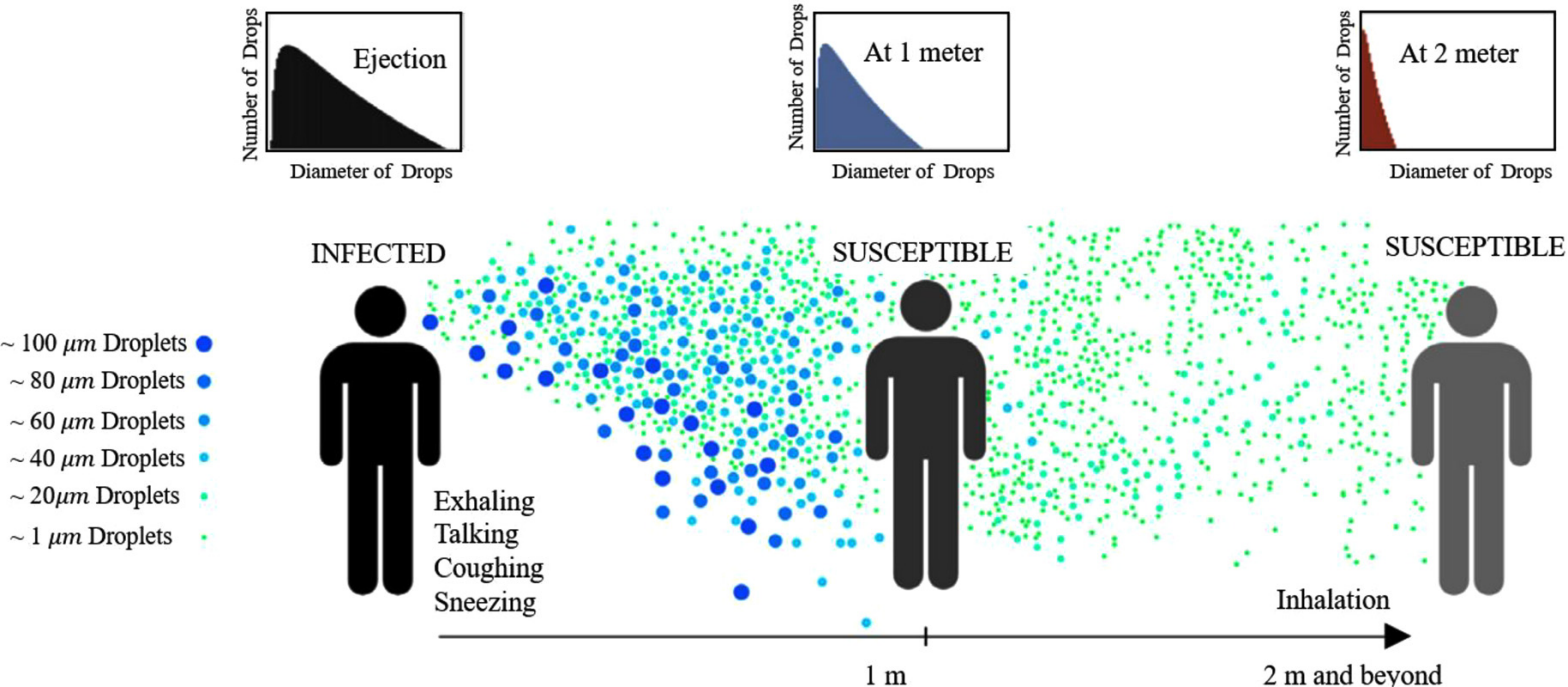
The two main transmission mechanisms:^26^ direct transmission by larger droplets and indirect airborne transmission by smaller airborne droplets. Adapted with permission from Balachandar *et al.*, Int. J. Multiphase Flow **132**, 1 (2020). Copyright 2020 Author(s), licensed under a Creative Commons Attribution (CC BY) license.

Balachandar *et al.*^26^ provided a quantitative analysis of the virus particle production, transport, and inhalation process. They have proposed a mathematical model for the initial PSD and the puff plume velocity profile. The schematic of the particle distribution presented in the study of Balachandar *et al.*^26^ is shown in Fig. 3. As can be seen, their modeling work indicated that direct transmission is mostly associated with larger droplets with diameter 60 *μ*m and larger, while smaller droplets with diameter less than 60 *μ*m may become airborne and lead to the potential for indirect transmission.

In some studies, particle dispersion has been mathematically modeled in more details.^27^ To help understand the above general observations and the usual recommendation of 2-m social distance (this safe social distance may depend on the local climate and the season^28^), we present here a very simple model capable of revealing some important features. To a good approximation, we can treat small contaminated droplets such as respiratory droplets as solid particles. Suppose that a droplet is released into the air at a height H with an initial horizontal velocity $u_{0}$. For simplicity, we assume the background air is moving at a horizontal mean velocity $u_{\mathrm{air}}$ (i.e*.,* the wind speed) in the same direction and neglect any vertical motion of the background air or the effect of the boundary layer near the ground. Using the typical empirical nonlinear drag^29^ and including the droplet inertia, gravity, and buoyancy force, the equations of motion for the droplet in the horizontal and vertical directions are, respectively,

$$\rho_{p}\frac{\pi d_{p}^{3}}{6}\frac{dU}{dt}=3\pi d_{p}\mu_{\mathrm{air}}\left(u_{\mathrm{air}}-U\right)f\left(Re_{p}\right),$$ (1)

$$\rho_{p}\frac{\pi d_{p}^{3}}{6}\frac{dV}{dt}=-3\pi d_{p}\mu_{\mathrm{air}}Vf\left(Re_{p}\right)+\left(\rho_{p}-\rho_{\mathrm{air}}\right)\frac{\pi d_{p}^{3}}{6}g,$$ (2)

where the nonlinear drag correction factor to the Stokes drag is^29^

$$f({Re}_{p})\equiv 1+0.15.{\left[\frac{\rho_{\mathrm{air}}d_{p}\sqrt{V^{2}+{\left({\bar{u}}_{\mathrm{air}}-U\right)}^{2}}}{\mu_{\mathrm{air}}}\right]}^{0.687},$$ (3)

where U and V are the droplet horizontal and vertical velocities, respectively, $d_{p}$ is the droplet diameter, $\mu_{\mathrm{air}}$ is the air dynamic viscosity, $\rho_{\mathrm{air}}$ is the air density, $\rho_{p}$ is the droplet density, and g is the gravitational acceleration. The value of ${\bar{u}}_{\mathrm{air}}$ (mean velocity of background air in a room) depends on the inlet velocity of a room (for example, the applied velocity by the ventilation system). The values of mean air velocity ${\bar{u}}_{\mathrm{air}}$ and $u_{\mathrm{rms}}$ are estimated as follows:^30^

$${\bar{u}}_{\mathrm{air}}=0.75\;u_{\mathrm{inlet}},$$ (4)

$$u_{\mathrm{rms}}=0.8\;{\bar{u}}_{\mathrm{air}},$$ (5)

where $u_{\mathrm{inlet}}$ is the vent or door inlet velocity and $u_{\mathrm{rms}}$ is the root mean square fluctuation velocity of the air turbulence.

Due to the nonlinear drag, the above two equations are coupled. To obtain approximate analytical solutions, we shall only include the nonlinear drag correction in the vertical direction so that the terminal velocity can be properly estimated. Namely, the particle Reynolds number is simplified as

$$Re_{p}\approx \frac{\rho_{\mathrm{air}}d_{p}V}{\mu_{\mathrm{air}}}.$$ (6)

Then, the vertical equation of motion can be written as^29^

$$\frac{dV}{dt}=-\frac{V}{\tau_{p}}+W,$$ (7)

where W is the terminal settling velocity, which can be computed by solving the following nonlinear equation:^29^

$$W\left(1+0.15\cdot {\left[\frac{Wd_{p}}{\nu_{\mathrm{air}}}\right]}^{0.687}\right)=\left(\frac{\rho_{p}}{\rho_{\mathrm{air}}}-1\right)\frac{d_{p}^{2}}{18\nu_{\mathrm{air}}}g.$$ (8)

Here, $\nu_{\mathrm{air}}\equiv \mu_{\mathrm{air}}/\rho_{\mathrm{air}}$ is the air kinematic viscosity. The response time $\tau_{p}$ is computed by^29^

$$\tau_{p}=\frac{\tau_{p,S}}{\left(1+0.15\cdot {\left[\frac{\rho_{\mathrm{air}}Wd_{p}}{\mu_{\mathrm{air}}}\right]}^{0.687}\right)},$$ (9)

where $\tau_{p,S}$ is the Stokes response time defined as $\tau_{p,S}=\rho_{p}d_{p}^{2}/(18\mu_{\mathrm{air}})$. Assuming the following conditions for the room air at 20 °C and 1 atm, $\mu_{\mathrm{air}}=1.825\times 10^{-5}\;\mathrm{kg}/(m.s)$, $\rho_{\mathrm{air}}=1.204\;\mathrm{kg}/m^{3}$, and $g=9.8\;m/s^{2}$, $\rho_{p}=1000\;\mathrm{kg}/m^{3}$, the values of W and $\tau_{p}$ are listed in Table II.

**TABLE II.** Properties of droplets after the nonlinear drag correction.

$d_{p}(\mu m)$	$\tau_{p,S}(s)$	W(cm/s)	$\tau_{p}$(s)	$Re_{p}$	$f\left(Re_{p}\right)$
10	0.0003	0.30	0.0003	0.002	1.341
20	0.0012	1.18	0.0012	0.016	1.548
30	0.0027	2.63	0.0027	0.052	1.724
40	0.0049	4.61	0.0047	0.122	1.883
50	0.0076	7.07	0.0072	0.233	2.029
60	0.0110	9.95	0.0102	0.394	2.166
70	0.0149	13.21	0.0135	0.610	2.297
80	0.0195	16.78	0.0171	0.885	2.422
90	0.0247	20.61	0.0210	1.224	2.542
100	0.0304	24.66	0.0252	1.627	2.658
110	0.0368	28.89	0.0295	2.096	2.771
120	0.0438	33.25	0.0339	2.633	2.880

Since the droplet response time shown in Table II is much less than the estimated sedimentation time W/H, namely, $\tau_{p}\frac{W}{H}\ll 1$, we further neglect the initial acceleration phase in the vertical direction, namely, we simply assume that the droplet settles at a constant velocity W.

Under all the above approximations, the equation of motion in the horizontal direction can now be written as

$$\frac{dU}{dt}=\frac{{\bar{u}}_{\mathrm{air}}-U}{\tau_{p}}.$$ (10)

It follows then the horizontal distance D that the droplet can travel in the air before reaching the ground is:

$$D=\frac{u_{a}H}{W}+\left(u_{0}-{\bar{u}}_{\mathrm{air}}\right)\tau_{p}\left[1-\text{exp}\;\left(-\frac{H}{W\tau_{p}}\right)\right]\approx \frac{u_{a}H}{W}+\left(u_{0}-{\bar{u}}_{\mathrm{air}}\right)\tau_{p}.$$ (11)

**FIG. 4.**
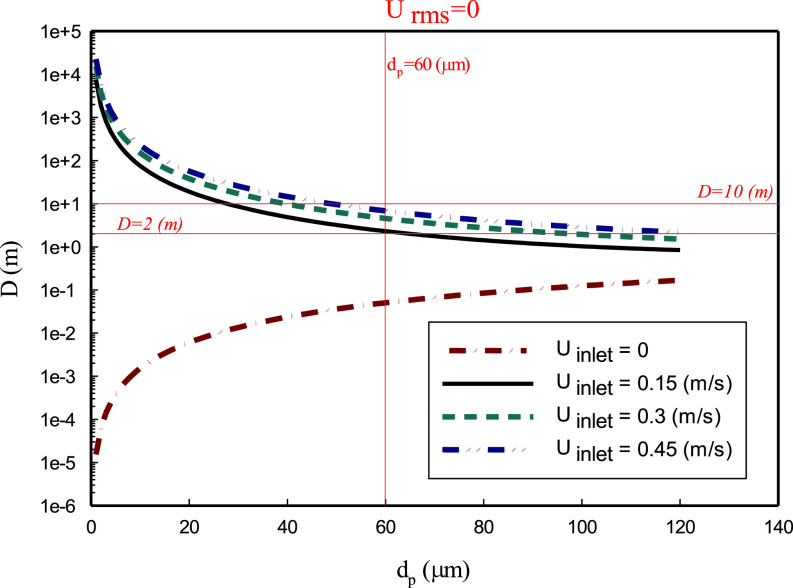
Effect of mean velocity of air on the distance traveled by droplets. The parameters used are: H=2 m, $u_{0}=5\;m/s$.

In Fig. 4, the horizontal distance D traveled by the droplet as a function of $d_{p}$ is shown. Several important observations can be made. First, without the room air motion, the distance is essentially ${\;u}_{0}\tau_{p}$, namely, the droplet's initial horizontal velocity will be reduced to zero due to the drag force over a time (the order of $\tau_{p})$, and then the droplet settles vertically afterward. Therefore, for a given initial horizontal velocity, larger droplets will travel more distance due to the larger inertial response time. For the range of droplet sizes ( $d_{p}<120\mu m$) considered, the maximum distance in the absence of air flow is less than 0.2 m, thus a social distance of 2 m is more than adequate. On the other hand, when there is a background airflow, the droplet velocity changes quickly from $u_{0}$ to ${\bar{u}}_{\mathrm{air}}$. Although ${\bar{u}}_{\mathrm{air}}<u_{0}$, the sedimentation time H/W is much larger than $\tau_{p}$, which causes the air velocity to play a significant role in the distance traveled by the droplet, and its effect on the traveled distance is more dominant than the initial velocity of the droplet [namely, the first term in Eq. (11), not from the droplet initial velocity]. Furthermore, smaller droplets, because of their larger sedimentation time, can travel a longer distance in the moving air, which is qualitatively different from the stagnant air case. In terms of social distance requirement, it is clear that 2 m is not adequate for small droplets. Interestingly, the distance traveled by droplets larger than 60 *μ*m is about 2 m and increases somewhat with the background airflow speed. Another interesting observation from the simple analysis is that the distance is very sensitive to the droplet size when $d_{p}<60\mu m$. For example, the traveled distance is always less than 10 m for $d_{p}>60\mu m$ in all cases, but it can easily reach over 100 m when droplets of $d_{p}<20\mu m$ are released in the airflow.

Next, we consider the effect of turbulent dispersion on the horizontal transport of droplets. We first note that the Lagrangian integral timescale $T_{L}$ of air turbulence is usually much larger than the response time. An estimate of $T_{L}$ could be

$$T_{L}∼\frac{0.1L_{\mathrm{inlet}}}{u_{\mathrm{rms}}}∼\frac{0.1\;m}{0.1\;\left(\frac{m}{s}\right)}∼1\;s,$$ (12)

where $L_{\mathrm{inlet}}$ is the length scale of the ventilation inlet (such as air vent, door, or window) and $u_{\mathrm{rms}}$ is the root mean square velocity of background air. Under this assumption, small droplets can be modeled as a passive scalar. Furthermore, the sedimentation time H/W is larger than $T_{L}$, and the mean square horizontal displacement can be approximated by the long-time theory of Taylor dispersion as^31^

$$\left\langle D^{2}\right\rangle =2u_{\mathrm{rms}}^{2}T_{L}t=2u_{\mathrm{rms}}^{2}T_{L}\frac{H}{W}.$$ (13)

**FIG. 5.**
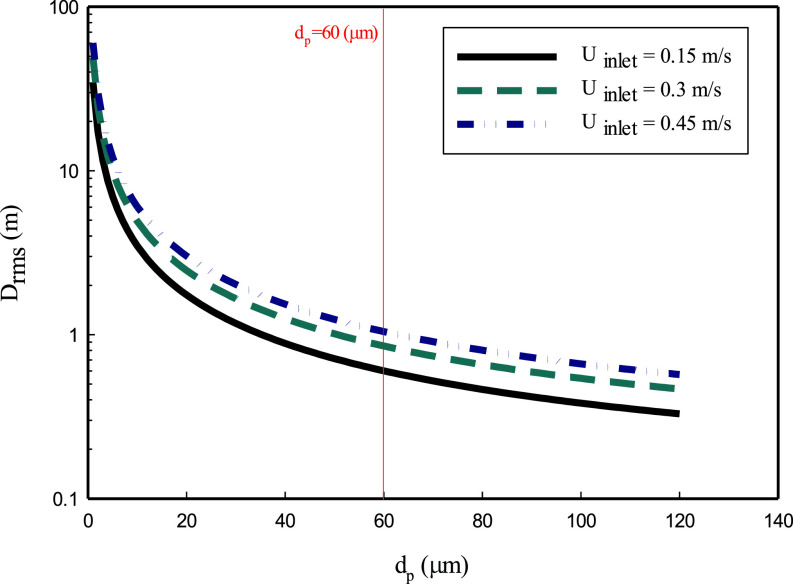
Effect of turbulent fluctuation motion on the distance traveled by droplets. The parameters used are: $\;H=2\;m,\;u_{0}=5\frac{m}{s},\;\mathrm{and}\;L_{\mathrm{inlet}}=1\;m$.

Therefore, the horizontal displacement of a droplet due to the turbulent fluctuation motion can only be estimated as^31^

$$D_{\mathrm{rms}}=\sqrt{D^{2}}=\sqrt{0.2\;u_{\mathrm{rms}}^{2}\frac{H}{W}\frac{L_{\mathrm{inlet}}}{u_{\mathrm{rms}}}}\;∼\sqrt{0.2\;u_{\mathrm{rms}}.L_{\mathrm{inlet}}\frac{H}{W}\;},$$ (14)

where the subscript *rms* denotes the root-mean-squared dispersion distance. To show the effect of a fluctuating velocity component on the distance traveled by the particle, the dispersion distance due to turbulent fluctuations alone is shown in Fig. 5. The fluctuation velocity in a simple room is considered based on the literature: $U_{\mathrm{rms}}=0.6\;U_{0}$, where $U_{0}$ is the mean inlet velocity.^30^ Comparing the distances traveled by the fluctuating velocity component and mean air velocity shows that the fluctuating velocity has less effect on the droplet displacement. However, according to Eq. (5), as the inlet velocity increases, the fluctuating velocity component also increases. Based on Fig. 5, as the fluctuating velocity component increases, the distance traveled by the droplets increases. Depending on the diameter, droplets larger than 60 μm travel less than 2 m, and particles smaller than 10 μm can travel up to 100 m. As the particle terminal velocity increases with the droplet diameter, the vertical motion due to the gravity force overcomes the motion due to the fluctuating velocity component, causing the larger particles to settle to the ground surface earlier.

We may now combine the effects of mean air motion and fluctuating air motion, by adding the two contributions together as an estimate for the worst-case scenario to model the total horizontal displacement as

$$D_{\mathrm{total}}=D+D_{\mathrm{rms}}=\frac{u_{a}H}{W}+\left(u_{0}-u_{a}\right)\tau_{p}+\sqrt{0.2\;u_{\mathrm{rms}}.L_{\mathrm{inlet}}\frac{H}{W}\;}.$$ (15)

This result shows that the total distance traveled by a particle in the horizontal direction depends on the droplet size, air flow mean velocity, and the level of turbulent fluctuations as measured by $u_{\mathrm{rms}}$, as well as the release height H, among other room and ventilation properties.

From the above simple analysis, we conclude that the two most critical parameters affecting the distance traveled by a droplet are the mean air velocity and droplet diameter. Although the fluctuating velocity component can carry the small droplets, the effect of the mean air velocity on the droplet transport is more significant. It is cautioned that the assumption of uniform background mean airflow is often not appropriate for an enclosed space such as a hospital room, and in such a case, a 3D numerical simulation must be executed to model the dispersion process.

## III. DROPLET SIZE DISTRIBUTION FROM HUMAN RESPIRATORY ACTIVITIES

Based on the discussions in Sec. II, it is found that the size of the droplets realized from human respiratory activity has a significant effect on their travel distance and, thus, the potential transmission range of airborne viruses. In a numerical simulation of the dispersion of respiratory droplets, the droplet size at the injection location is needed as the boundary conditions for a CFD simulation. As noted in Sec. II, the droplet size affects the droplet's terminal velocity and inertial response time and, consequently, the interactions of droplets with the air flow. Therefore, accurate determination of the droplet size distribution when released to the air is essential for studying the spread of airborne viruses, their near-reality modeling, and design of appropriate ventilation systems. This section reviews the literature reporting experimental data of the size distributions of respiratory droplets, in order to suggest a reliable distribution to use in a CFD model.

Many experimental studies on the size of respiratory droplets of healthy^20^ or infected^32^ subjects have been performed using different devices, some of which are mentioned in this section. Gralton *et al.*^17^ and Han *et al.*^33^ reviewed the experimental studies of particle size from 1899 to 2011. Based on these review papers, there are significant inconsistencies in the existing data, and the reported particle sizes could vary between a few millimeters and less than one micrometer. These inconsistencies in the measured data on the PSD mainly come from two sources. The first is the natural variations among different human individuals.^33^ A person's cough may vary with gender, weight, and other individual characteristics; even various coughs from one person during a day may have different features (such as the peak velocity, size distribution, and velocity profile). The second is the variations of the accuracy and detectable size range of measurement devices.^33^

In addition to the measured PSD, parameterizing the size distribution with minimum error is essential. The lognormal distribution is one of the most popular descriptions used to parameterize the aerosol size distribution, in which the probability distribution of $\log d_{p}$ is assumed to be normal.^34^ This type of distribution method can easily cover a wide range of particle sizes. Even if the size range is narrow, the lognormal distribution can approximate a normal distribution. Under this description, the PSD is defined by a concentration distribution using a logarithmic size axis, as follows:^34^

$$\frac{dN}{\mathrm{dlog}d_{p}}=\frac{dN}{\log d_{p,u}-\log d_{p,l}}.$$ (16)

Here, dN is the particle concentration for a size bin defined as $\log d_{p,u}\leq \log d_{p}\leq \log d_{p,l}$, where $d_{p,u}$ and $d_{p,l}$ are the upper and lower diameter boundary of the size bin, respectively.

Although accurate determination of the size distribution of droplets released from the respiratory activity is a challenging task,^35^ valuable experimental results have been reported. In this part of the review article, some important results about PSD are mentioned. It is noteworthy that researchers have focused more on measuring the size of cough droplets, while data on the sneezing particle size are scarce.^17^

The range of droplet's initial size varies significantly in the literature. For example, based on the results of Duguid,^20^ during coughing and sneezing of healthy subjects, the diameters of most droplets produced from the mouth are around 10 *μ*m, and some are even larger than 100 *μ*m, whereas Lee *et al.*^36^ reported the coughing particles size of the young and healthy adults being less than 100 nm. These different reported ranges could be due to different instruments/devices used to measure the PSD, as each instrument may be limited to a certain range of size. Yang *et al.*^37^ measured the size distribution of coughed droplets and droplet nuclei from 54 healthy test subjects. They found a significant difference in droplet size depending on the sex in three age groups. Based on their results, the 30–50-year-old age group produced the highest aerosol concentration, and there was also a higher airborne droplet concentration by males than females. According to their results, the size of coughed droplets varied between 0.62 and 15.9 *μ*m, and the average diameter was 8.35 *μ*m.

Han *et al.*^33^ measured the droplet size during sneezing by a laser particle size analyzer. To reduce the effect of droplet evaporation on the results, they performed measurements right after the mouth. Based on the results, the number of particles with a diameter of fewer than 20 μm is deficient, and particles smaller than 1 μm were not reported at all. They pointed out that for smaller diameters, the results may not be accurate enough due to the used measurement system. They showed that the geometric mean diameter of all the sneeze droplets is 360.1 ( μm) for unimodal distribution and 74.4 ( μm) for bimodal distribution. Unimodal and bimodal mean that the PSD has one peak and two peaks of diameter, respectively.

Papineni and Rosenthal^38^ compared droplet deposition analysis (DDA) with optical particle counter (OPC) direct measurements. They characterized the size distribution of droplets exhaled by healthy individuals during four respiratory actions (mouth breathing, nose breathing, coughing, and talking). The results from the two methods are inconsistent, but they show that 80%–90% of particles from human expiratory activities were aerosols with the diameter smaller than 1 *μ*m. Morawska *et al.*^39^ conducted a comprehensive experiment to analyze the PSD during coughing, breathing, and talking. They show that the PSDs composed of one or two modes, where the majority of them were produced with diameters below 0.8 μm (average concentrations up to 0.75 cm^3^). The second mode was reported at 1.8 μm (with a lower concentration of up to 0.14 cm^3^). They mentioned that the additional particles in a diameter of 3.5–5 μm produced during sustained vocalization due to the aerosolization of secretions lubricating the vocal chords. The minimum achievable distance was 10 mm in their experimental setup, and the droplets reached the measurement region within approximately 0.8 s. They cannot detect the non-equilibrium droplet evaporation for particles between 0.5 and 20 μm, indicating the equilibrium droplet size occurred within 0.8 s.

Chao *et al.*^40^ examined the droplet size distribution of coughing and speaking over healthy subjects, and also they investigated the velocity profile of the expired jet close to the mouths. They measured the droplet size and expired jet velocity by interferometric mie imaging (IMI) and the PIV (Particle Image Velocimetry) method, respectively. In their experimental setup (measuring immediately at the mouth opening), the effect of evaporation on the droplet size distribution is negligible. They pointed out that previous studies did not make the effort to measure the droplet size right at the mouth/nose exit. Based on their results, at the 10 mm distance, the mean diameter of the droplets from coughing and speaking was 13.5 and 16 *μ*m, respectively. Their results show that the mean diameter at a distance of 60 mm, compared to 10 mm, decreased modestly. It might be caused by evaporation of the particles or the less traveling time of small particles. Measurement of the exhaled jet shows that the initial velocity of coughing and speaking at a distance of 60 mm is 11.7 and 3.9 m/s, respectively. They have compared their results with the results of Duguid^20^ and Loudon and Roberts^21^ and showed that their results are more consistent with Duguid^20^ for large droplets. They mentioned that the IMI technique could capture mostly large droplets because of the limitations and uncertainties.

Zayas *et al.*^41^ examined the PSD of droplets produced during coughing of healthy participants by the laser diffraction system. The weight, height, and age of the 26 male and 19 female participants ranged 53–77 kg, 159–177 cm, and 19–49 ages, respectively.^41^ Contrary to the other mentioned references, their results showed that 97% of droplets in a single cough are smaller than 1 *μ*m. Due to the variety of sex, height, weight, and ages of the participants, they concluded that the effect of age, weight, height, and gender on the size distribution is not significant.

**FIG. 6.**
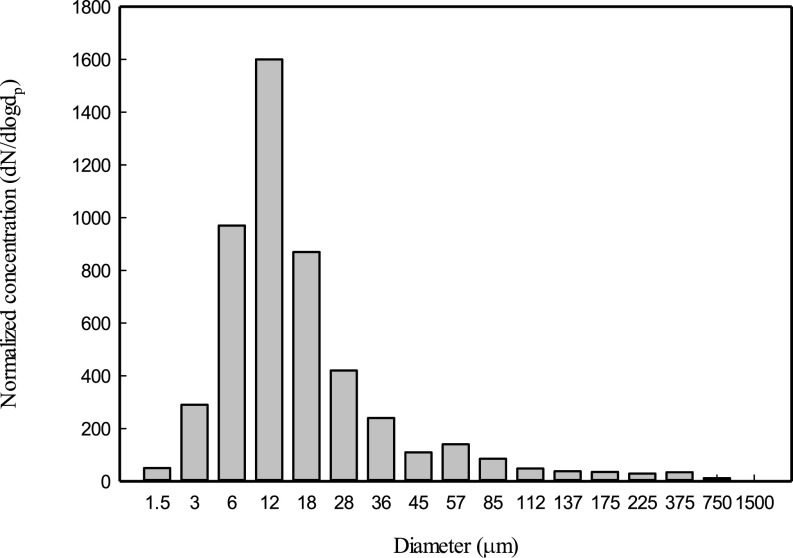
The normalized concentration—experimental results of Ref. 20. Adapted with permission from J. P. Duguid, J. Hygiene **44**, 471 (1946). Copyright 2020 Author(s), licensed under a Creative Commons Attribution (CC BY) license.

Duguid^20^ studied the size distribution of the initial droplets produced during speaking, sneezing, and coughing, using impaction plates placed in front of the subject's mouth.^20^ They showed that particles due to the respiratory activity range from 1 to 2000 μm in diameter. According to their results, 95% of the droplet's diameter range from 2 to 100 μm. Their results showed that the droplet's predominant size is in the range of 4–8 *μ*m. Figure 6 shows the normalized PSD (Eq. 16). As illustrated in Fig. 6, the peak concentration occurs for the diameter range between 6 and 18 μm. Based on the terminology in Table I, it can be concluded that the concentrations of droplet nuclei and small droplets are significant in the investigation of Duguid.^20^

Xie *et al.*^42^ examined the PSD and the mass of talking and coughing droplets by water-sensitive wipes and a microscope. They used three men and four women for testing and compared results with those of Duguid^20^ and Loudon and Roberts.^21^ In Table III, the results of Xie *et al.*^42^ are compared with the other two references. As seen, they are inconsistent. One of the reasons for the difference in results can be the various methods of sampling and measurement. Xie *et al.*^42^ used a box, whereas Duguid^20^ used a plate in front of the mouth for sampling. Although the mentioned studies have focused on healthy participants, Fennelly *et al.*^32^ showed that the individuals with influenza cough out a greater volume of aerosol particles than they do when they are healthy. Overall, the results of different studies have shown that the diameter of particles emitted from infected humans is larger than that of healthy individuals.

**TABLE III.** Peak diameter range during coughing and talking—Xie *et al.*,^42^ Duguid,^20^ and Loudon and Robert.^21^

	Peak diameter range ${d\;}_{p}(\mu m)$		
	Xie *et al.*^42^	Duguid^20^	Loudon and Roberts^21^
Talking	$35<d_{p}<50$	$15<d_{p}<25$	$8<d_{p}<100$
Coughing	$35<d_{p}<100$	$15<d_{p}<25$	$22<d_{p}<73$

**FIG. 7.**
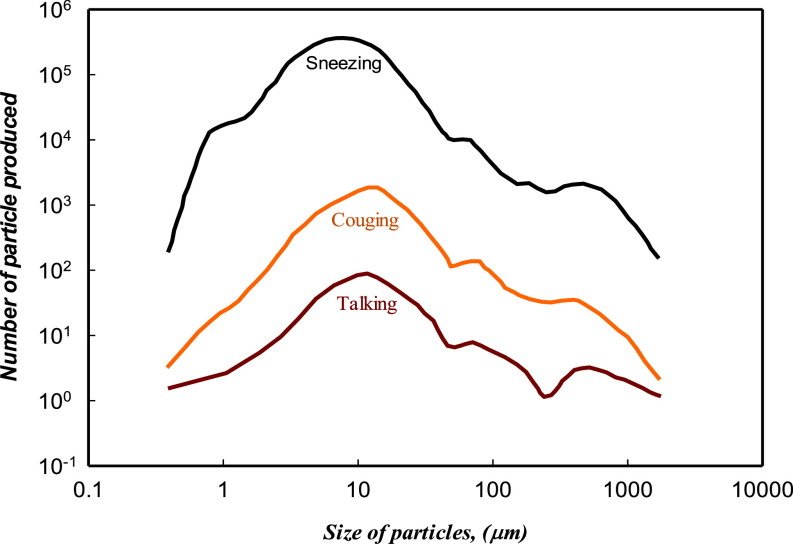
Particles size distribution emitted during talking, coughing, and sneezing^40^ based on Ref. 20. Adapted with permission from J. P. Duguid, J. Hygiene **44**, 471 (1946). Copyright 2020 Author(s), licensed under a Creative Commons Attribution (CC BY) license.

In addition to the particle size range, the number of particles in each size is also important. In some references,^10,43^ the size ranges in terms of the particle diameter caused by coughing and talking are the same; however, as seen in Fig. 7, the number of particles generated by coughing is more than that by talking;^10,43^ also based on Fig. 7, the particle number emitted during sneezing is more considerable than coughing and talking.

It should be noted that the diameter size of viruses has been studied in the literature. Lindsley *et al.*^44^ measured influenza virus in droplet nuclei generated by a patient during coughing. They reported that 42% of detected viruses were found in droplet nuclei with a diameter less than 1 *μ*m, 23% in droplet nuclei from 1 to 4 *μ*m, and 35% in droplet nuclei > 4 *μ*m.^44^

In addition to accurately measuring the PSD of the respiratory activities, mathematical modeling of the PSD is also essential. It seems necessary to study the fitting capability of different distribution models to provide a PSD description for respiratory droplets. In most numerical studies, the Rosin–Rammler distribution^45^ has been used to model the initial PSD. The Rosin–Rammler distribution function is based on the assumption that an exponential relationship exists between the droplet diameter $d_{p}$ and the mass fraction of droplets $Y_{d}$ with diameter greater than $d_{p}$, as follows:^45^

$$Y_{d}=e^{-{\left(\frac{d_{p}}{\bar{d}}\right)}^{n}},$$ (17)

where $\bar{d}$ refers to the mean diameter and n, the spread parameter. In the recently published numerical investigations^46,47^ based on the experimental data,^42^ the mean diameter and the spread parameter were assumed to be 80 μm and 8, respectively.

## IV. BOUNDARY CONDITIONS FOR SIMULATING THE AIRFLOW FROM BREATHING, COUGHING, AND SNEEZING

Based on what discussed above, applying the correct initial and boundary conditions in a numerical simulation is very important. Boundary conditions and the initial condition far from reality lead to incorrect results. In simulating the droplets dispersion caused by the human respiratory activity, several important input parameters are needed and they include:

• mouth or nose opening area,
• flow rate and duration of the respiratory event,
• time-dependent velocity profile during the event, and
• temperature profile during the event.

While inhalation from both mouth and nose is aerodynamically the same, the flow patterns of the exhaled air from mouth and nose are entirely different;^19^ thus, the boundary conditions for simulation of the produced jet from the mouth and nose are different.

In a numerical simulation, creating a geometric model is one essential step. If the details are considered in the geometric model, the problem will be simulated closer to reality. The geometric shape of the mouse and nose may be a cause for concern in simulating respiratory activity and attracted the researchers' attention. In their review paper, Ai and Melikov^25^ presented the different mouth, nose, and opening area geometries considered in the previous investigations. Three shapes for the mouth opening, namely, semi-ellipsoid, circular, and ellipsoid were considered, while nostril openings were all circular. The area of the mouth opening ranged from 100 to 123 mm^2^ during normal breathing but exceeded 300 mm^2^ during coughing. These geometrical differences between different studies contributed partially to the discrepancies in the simulated transport of airborne aerosols. Here, we instead focus on the time characteristics of the exhaled jet velocity without attending to the shape of the opening area.

**FIG. 8.**
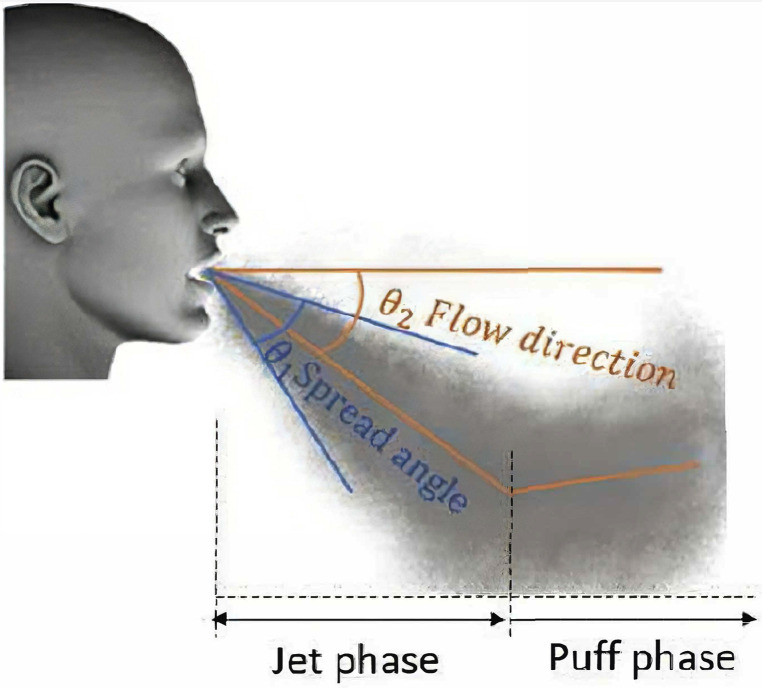
The flow direction and flow spread angle.^18^ Reprinted with permission from Merghani *et al.*, Indoor Air **31**, 7 (2021). Copyright 2021 Author(s), licensed under a Creative Commons Attribution (CC BY) license.

The created jet during breathing activities has features such as the flow direction, spread angle, and time-dependent velocity profile. Various values have been reported for the flow direction and spread angle.^17^ Figure 8 illustrates the definition of the flow direction and flow spread angle. As can be seen, the angle between the jet centerline and the horizontal direction is called the flow direction ${(\theta }_{2})$, and the angle between the plume boundaries is defined as the spread angle $\left(\theta_{1}\right)$. Therefore, adopting a constant horizontal velocity profile instead of time-dependent sneezing and the coughing jet profile leads to a horizontal jet that is far from reality and can affect particle trajectory and distort the accuracy of the numerical results. To explain more about the effect of injection angle on the contaminated distance during sneezing, we mention the numerical results of Pendar and Páscoa.^48^ They showed that the contaminated distance declines considerably by increasing the injection angle: increasing the sneeze injection angle from 3° to 43° caused the maximum contaminated distance to drop from 2.2 to 1.8 m (by about 22%). To set the correct boundary condition, the proposed velocity profile and puff characteristics of the respiratory activities in some investigations can be used. Simha and Rao^49^ presented the velocity profile with and without masks, and Kwon *et al.*^50^ suggested a relation between the height and horizontal velocity of coughing and speaking for male and female. Gupta *et al.*^19,22^ presented the mouth and nose opening area for female and male, and they stated the relationship between the respiratory frequency and the physiological parameters (body height and body weight) for the male and female subjects.

**FIG. 9.**
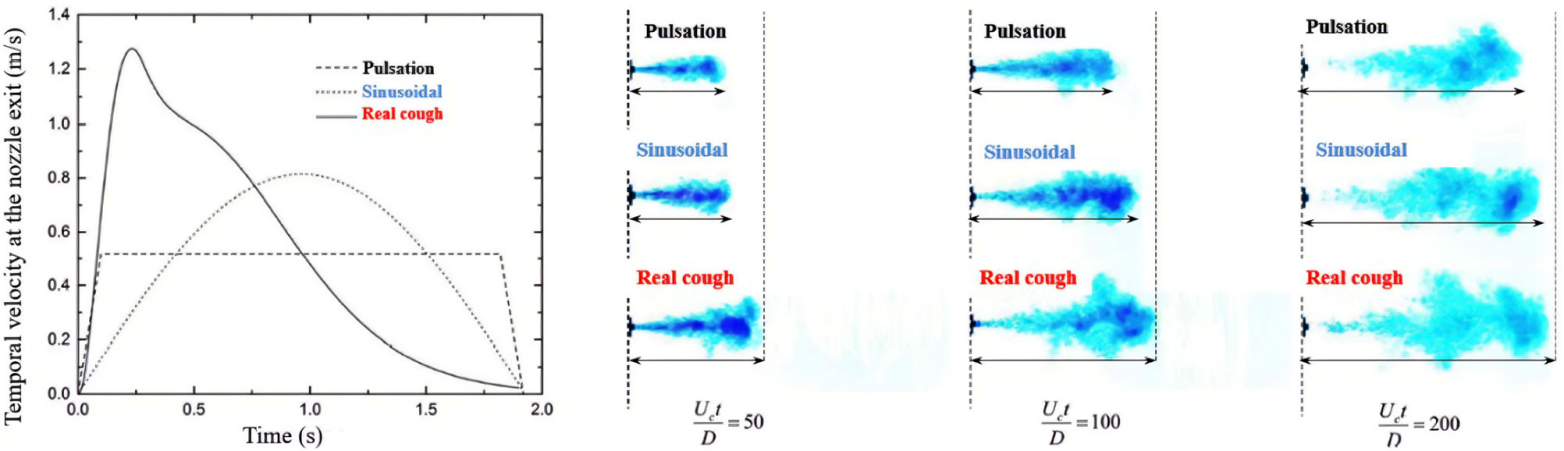
Three temporal profiles at the nozzle exit and the effect of the exit velocity profile on cough cloud dispersion.^51^ Reprinted with permission from J. Wei and Y. Li, PLoS One **12**, 0169235 (2017). Copyright 2017 Author(s), licensed under a Creative Commons Attribution (CC BY) license.

The penetration length of the cough strongly depends on the type of the applied boundary conditions. Here, the penetration length is defined as the distance traveled by the jet when the air velocity drops below 0.01 m/s.^51^ Before mentioning the characteristics of the jet profile, we want to show the effect of the velocity profile on the penetration length of the exhaled jet. Wei and Li^51^ examined the effect of three temporal velocity profiles including two simple profiles (pulsating, sinusoidal) and a real-cough (based on the experimental data of Gupta *et al.*^22^) on the penetration length of cough. Their tests were conducted in water and had some limitations, that is, the realistic mouth opening is not circular. In Fig. 9, the three temporal profiles and the visualization of cough clouds are shown. According to the velocity profiles, the peak velocity during the starting-jet stage for the sinusoidal profile and real cough is $1.57U_{c}$ and $2.94U_{c}$, respectively. $U_{c}$ is the average injection velocity^51^

$$U_{c}=\frac{1}{t_{\mathrm{inj}}}\int_{0}^{t_{\mathrm{inj}}}U\left(t\right)dt,$$ (18)

where $t_{\mathrm{inj}}$ is the cough duration.

Figure 9 shows that the temporal velocity profile significantly affects the dispersion of cough clouds and penetration length. As seen, during the starting-jet stage, the real cough's penetration length is longer than in the other two cases. The penetrated length of the real cough is more than two others velocity profiles due to the larger maximum velocity. However, dispersions of cough clouds gradually have similar behavior because, over time, the initial momentum of the cough cloud disappears in the environment. The cough cloud can have a significant effect on individual droplet trajectories.

**FIG. 10.**
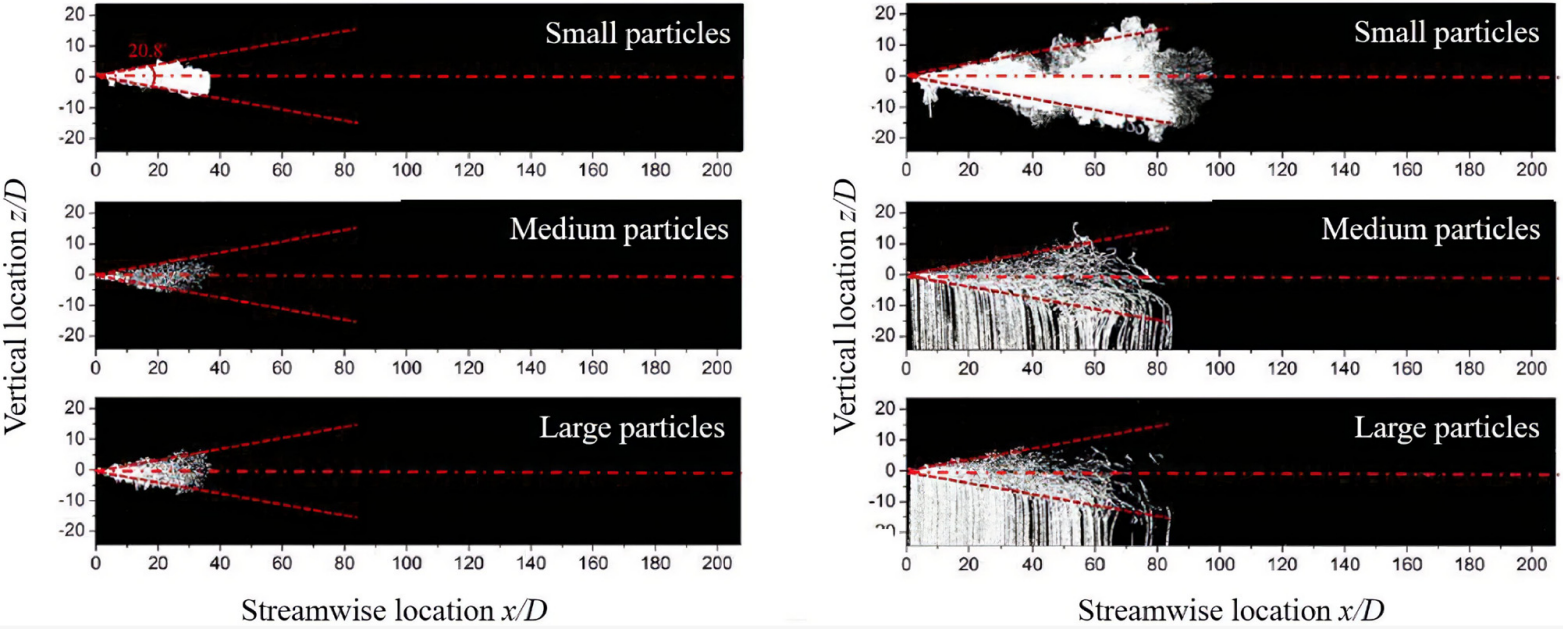
Particles dispersion pattern (pulsation velocity profile).^51^ Reprinted with permission from J. Wei and Y. Li, PLoS One **12**, 0169235 (2017). Copyright 2020 Author(s), licensed under a Creative Commons Attribution (CC BY) license.

In addition to the cough cloud characteristics, the particle size can affect the particle dispersion pattern. Compared to large droplets, smaller droplets are suspended by the buoyant cough cloud and transported over long distances.^52^ Wei and Li^51^ examined the effect of the particle size on the particles dispersion pattern (only for the pulsation velocity profile). The particle diameters ranged in $\left(30–50\;\mu m\right),\;\left(210–250\;\mu m\right)$, and $\left(355–470\;\mu m\right)$ and were classified as small, medium, and large particles, respectively. Figure 10 shows the particle dispersion pattern at the end of injection $\left(t=t_{\mathrm{inj}}\right)$ and $\left(t=10\;t_{\mathrm{inj}}\right)$. As we can see, at the end of injection $\left(t=t_{\mathrm{inj}}\right)$, the dispersion pattern of small, medium, and large particles is not significantly different and the maximum penetration length of particles is about 38 times D (D is the nozzle diameter). At $t=10\;t_{\mathrm{inj}}$, the small particles' dispersion pattern is very different from that of medium and large particles. Small particles remained within the jet while medium and large particles tend to escape the jet and settle on the ground due to the gravity force.^17^

One of the important characteristics of the discharged jet by the respiratory activity is the jet's maximum velocity. Depending on the location of breathing or coughing (from the mouth or nose), the maximum velocity of the discharged jet varies. The maximum velocity of the exhaled jet through the mouth breathing was reported to be 2–3 m/s.^43^ The velocity of the jet during nose breathing was reported to be 3 m/s.^53^ However, in some studies, the maximum speed is reported in the range of 1–2 m/s.^42^ This inconsistency in the literature makes it difficult to set the correct boundary condition in a numerical simulation. The velocity of the airflow exhaled by sneeze is much larger than that of breath and cough.^22,54^ Until now, studies on the sneeze velocity profile and PSD during sneezing are still rare.^17,55,56^

To formulate the velocity profile of respiratory activities and make it possible to set it as a boundary condition in a numerical simulation, we reviewed some investigations in this area.

By using PIV and the indoor acrylic chamber, Kwon *et al.*^50^ examined the initial velocity distribution of exhaled air from coughing and speaking of 17 males and 9 females. Their results showed that the initial velocity characteristics were affected by the gender of individuals. Based on the results, the average initial coughing velocity was 15.3 and 10.6 m/s, and the angle of the exhaled air from coughing was 38° and 32° for males and females, respectively. Their results showed that the average initial speaking velocity was 4.07 and 2.31 m/s, and the angle of the exhaled was 49° and 78° for men and females, respectively. They presented a linear relationship between the individual's height and the coughing and speaking velocity.

**FIG. 11.**
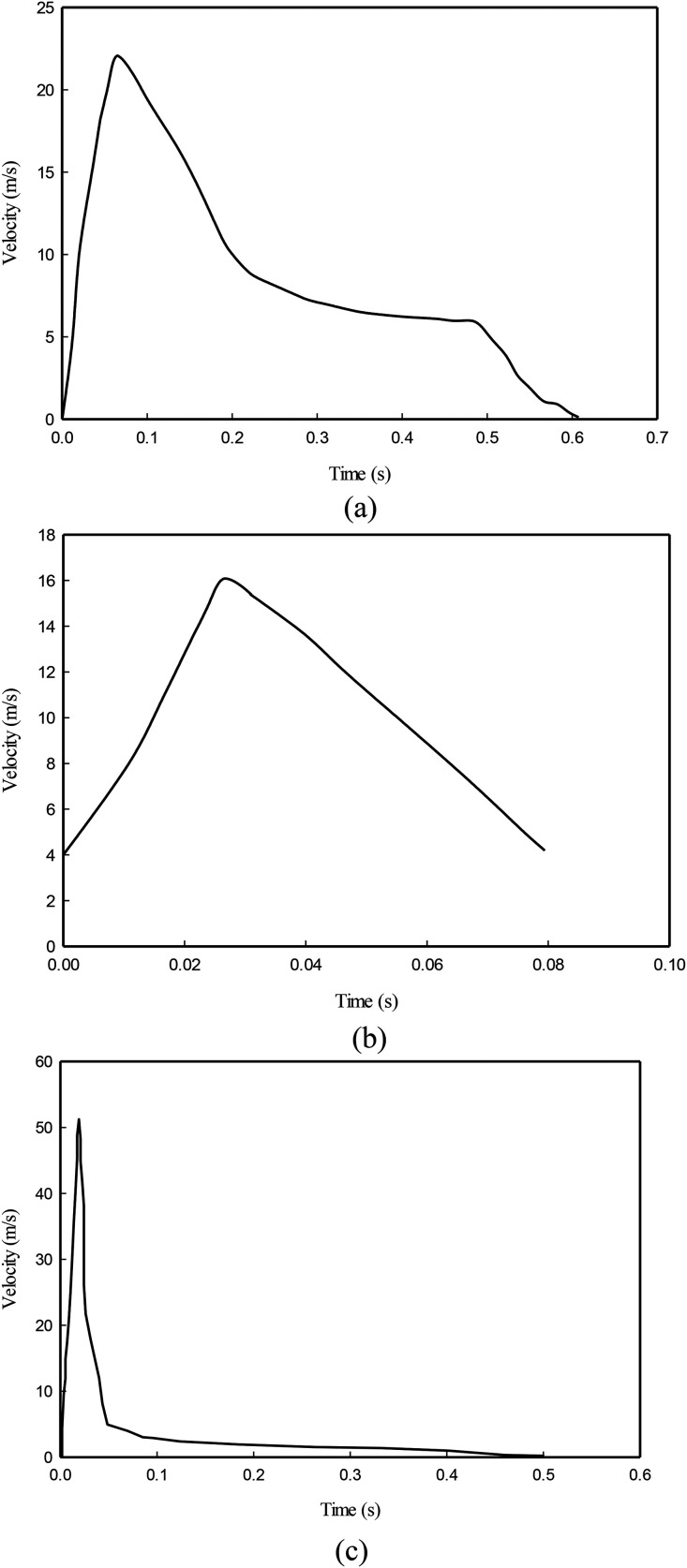
(a) Inlet transient velocity profile of coughing, Gupta *et al.*^22^ Adapted with permission from J. K. Gupta, C.-H. Lin, and Q. Chen, Indoor Air **19**, 517 (2009). Copyright 2020 Author(s), licensed under a Creative Commons Attribution (CC BY) license. (b) Inlet transient velocity profile of coughing.^22^ Reproduced from Wang *et al.*, “The motion of respiratory droplets produced by coughing,” Phys. Fluids **32**, 125012 (2020) with the permission of AIP Publishing. (c) Inlet transient velocity profile of sneezing,^57^ (based on Gupta *et al.*^22^). Reproduced from Fontes *et al.*, “A study of fluid dynamics and human physiology factors driving droplet dispersion from a human sneeze,” Phys. Fluids **32**, 111904 (2020) with the permission of AIP Publishing.

Gupta *et al.*^19^ characterized exhaled airflow from breathing and talking. The flow rate of breathing and talking over time was presented by a sinusoidal function and a constant, respectively. In contrast, the opening size and the direction of the exhalation jet varied among subjects; they could not fit on it a correlation as a function of the subject's physiological parameters. Gupta *et al.*^22^ examined the flow characteristics such as flow rate variation with time, the jet direction, and mouth opening area during coughing. Based on their results, although the jet direction and the mouth opening area during a cough seemed not related to the human subjects' physiological parameters, the flow rate variation during the time can be correlated as a function of the physiological parameters. The transient inlet cough velocity (a mouth diameter of D = 0.0217) is shown in Fig. 11(a). As seen, the jet flow lasts 0.61 s, and the velocity peaks 22.06 m/s as t = 0.066 s. Also, Gupta *et al.*,^22^ by employing the PIV method and the visualization of cough cloud, presented the cough velocity profile. Based on their results as shown in Fig. 11(b), the initial velocity at t = 0 s is ∼4.0 m/s and then rapidly increases to 15.0 m/s at t = 0.03 s. The velocity gradually reduces to 4.0 m/s at time t = 0.08 s. Based on the results of Gupta *et al.*,^22^ Fontes *et al.*^57^ presented the velocity profile [Fig. 11(c)] for sneezing and used it in a numerical simulation as a boundary condition for sneezing. As can be seen, the maximum velocity of sneezing is more than twice of coughing, so the plume of sneezing is completely different from coughing. The curve fitting of the presented velocity profiles of Figs. 11(a)–11(c) can be an accurate boundary condition in a numerical simulation.

As mentioned, for the initial velocity of the jets produced by respiratory activities, various mathematical models have been proposed based on experimental tests. Although the information presented in them, including the maximum speed and the time to reach the maximum velocity, are not well compatible with each other, all of them have shown that the velocity profile of coughing varies over time. Therefore, it is recommended that in future numerical simulations, the constant velocity model should not be applied as the boundary condition for coughing or sneezing.

Finally, in Table IV, important features and limitations of the previous numerical studies are noted. It helps us to identify which numerical studies considered PSD, modeled the droplet evaporation, provided validation against the experimental data, adopted time-dependent velocity profile at the mouth/nose, or considered the composition of real cough droplets. Furthermore, in Table V, we show specific details of the applied time-dependent velocity profile at the mouth/nose in the previous numerical simulations of the human respiratory activities. It is observed that the applied velocity conditions are not consistent in the literature. The numerical results of Bi^7^ showed that the time history of the inlet cough velocity has a significant effect on the particle dispersion pattern, although it does not significantly change the evaporation rate of the droplets.

**TABLE IV.** Details of representative numerical studies of droplet dispersion due to the respiratory activity.

Details of the numerical simulation	References
Initial PSD	Ref. 10 based on Ref. 20; Ref. 6 based on Ref. 33; Ref. 34 based on Ref. 58; Ref. 48; Ref. 59; Ref. 60 based on Ref. 42; Ref. 46; and Ref. 61
Validation with experimental data (pure water droplet)	Refs. 4, 14, 35, 62–65 and 62
Considering inlet velocity profile	Refs. 63, 66, 67, and 68 based on Refs. 6, 22, 61, 69, and 10 based on Refs. 70 and 20
Evaporating model	Refs. 4, 6, 10, 20, 34, 35, 46, 48, 52, 55, 59, 61–66, 71, 72, and 73–76
Only solid particle injection (not droplet and evaporation)	Refs. 62, 53, 77, and 78
Pure water as material	Refs. 4, 6, 10, 20, 34, 35, 46, 48, 55, 59, 63–65, 72, and 79
Real saliva as material	Refs. 62 and 61
Effect of environment condition on evaporation	Refs. 4, 10, 20, 35, 46, 55, 59, 61, 64, 65, and 62

**TABLE V.** Details of the applied boundary condition on the mouth/nose in the previous numerical simulations of the human respiratory activities. The velocity is in m/s and time in s unless stated otherwise.

Respiratory activity	The applied boundary condition
Breathing^63^	$v_{\;\text{sin}\;}=4.5\text{sin}\left(1.79\;t\right)\;$
	$V_{\mathrm{inlet}}=\left\{\begin{array}{c} 0,\;v_{\;\text{sin}\;}<0\\ v_{\;\text{sin}\;}\left(t\right),\;v_{\;\text{sin}\;}>0 \end{array}\right.$
Breathing^14^	Constant velocity: 1 m/s
Breathing^69^	$V_{\mathrm{inlet}}=\frac{V_{t}}{T_{b}A_{mo}}\pi \text{cos}\left[\frac{2\pi t}{T_{b}}-\frac{\pi }{2}\right]$ volume of breathing: $V_{t}=0.5\times 10^{-3}\;m^{3}$, the period of breaathing: $T_{b}=5\;s$, $A_{mo}$ is the area of the mouth opening during breathing: $3.5\times 10^{-4}\;m^{2}$, t is the time
Breathing^54^	Sinusoidal curve, with 17 times per minute with a time-mean rate of 8.4 L/min
Breathing^80^	Sinusoidal curve, with 10 times per minute with a time-mean rate of 6.0 L/min
Breathing^81^	Constant velocity: 0.107 m/s
Breathing^82^	Constant velocity: 0.2 m/s
coughing^83^	Constant velocity: 10 m/s
Coughing^66^	$V_{\mathrm{inlet}}=\left\{\begin{array}{ll} \frac{w_{m}}{t_{m}}t, & 0\leq t\leq t_{m}\\ w_{m}-\frac{w_{m}}{t_{c}-t_{m}}\left(t-t_{m}\right), & t_{m}<t<t_{c}\\ 0, & t>t_{c} \end{array}\right.w_{m}=4.8\frac{m}{s},\;t_{m}=0.15\;s,\;t_{c}=0.4\;s$
Sneezing^6^	Pressure profile: $P\left(t\right)=\frac{c_{1}t^{a_{1}-1}e^{\frac{-t}{b_{1}}}}{b_{1}^{a_{1}}\Gamma (a_{1})}+\frac{c_{2}t^{a_{2}-1}e^{\frac{-t}{b_{2}}}}{b_{2}^{a_{2}}\Gamma (a_{2})}\;\Gamma$ is the gamma function, $a_{1}$ = 4, $b_{1}$ = 0.0235 s, $c_{1}$ = 860.1073 Pa·s, $a_{2}$ = 9, $b_{2}$ = 0.028 s, and $c_{2}$ = 674.3917 Pa·s
Breathing, coughing, and sneezing^84^	Constant velocity: 1 m/s, 10 m/s, and 35 m/s were applied for breathing, coughing, and sneezing

## V. NUMERICAL SIMULATION OF THE EVAPORATION PROCESS

In this section, the literature related to the numerical simulations of respiratory droplet dispersion is reviewed. Type of the respiratory activities, description of initial size distribution, evaporation rate modeling, composition of the sputum droplets, environment condition, and some overall details about the numerical methods are discussed. A summary of the recent numerical papers is presented in Table IV. As seen in some numerical studies, there was no validation with any experimental data related to the real cough/sneeze droplets, although some of them validated their numerical results with literature data based on pure water droplets. The majority of the numerical studies in the literature have not been validated with experimental results, because experimental studies in this field could not provide the necessary details. Numerical simulation of a practical test case requires sufficient initial and boundary conditions, which are rare in experimental studies. An experimental study with full details of the problem's initial conditions is recommended as a benchmark for the future studies. Modeling the droplet's evaporation rate is the most critical step in the numerical simulation of the respiratory droplet dispersion. Based on our knowledge and the literature review (Table IV), majority of the numerical studies used Ranz and Marshall^85^ correlation to calculate the heat and mass transfer coefficient. In Sec. V A, the details and limitations of this correlation and the modified form of this correlation are discussed.

### A. Droplet evaporation model

**FIG. 12.**
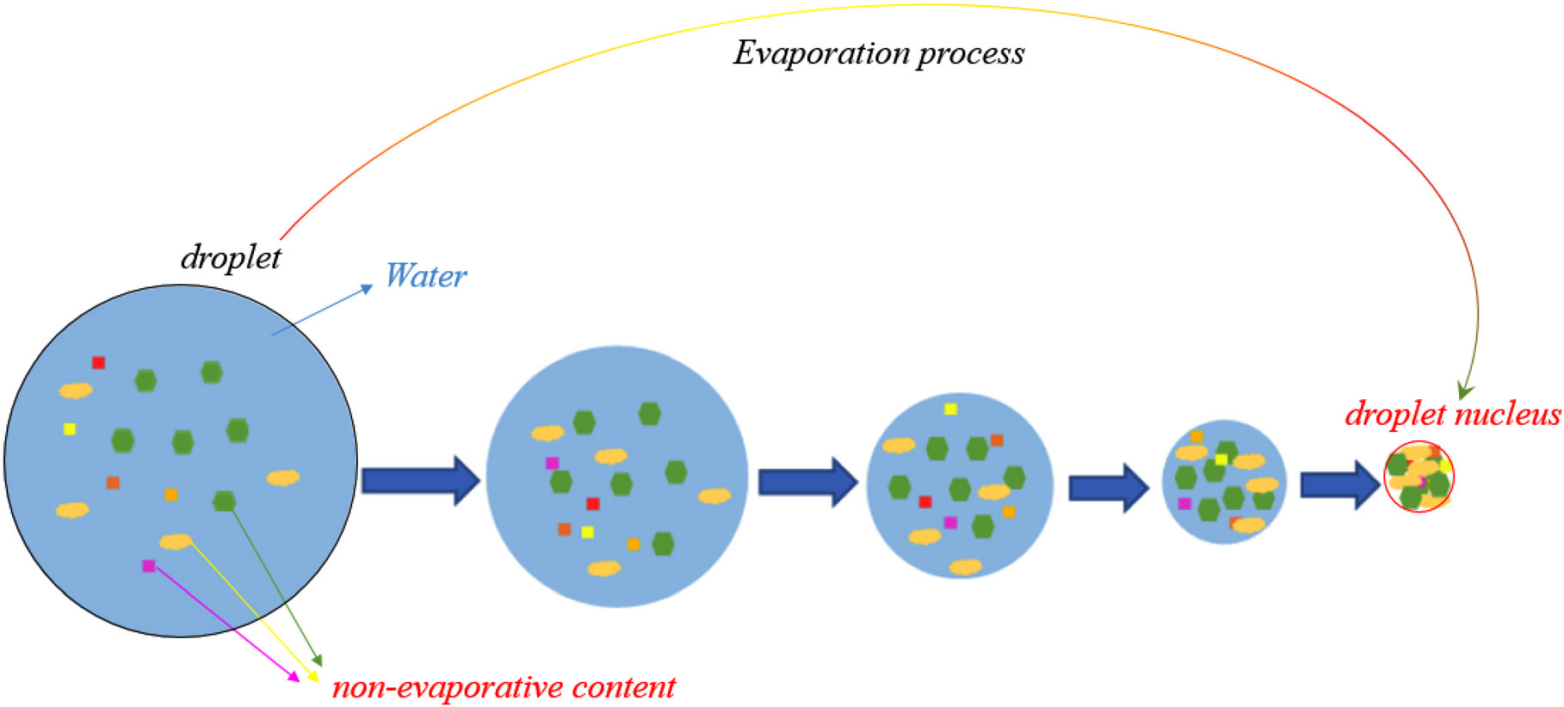
The evaporation process of a droplet and formation the droplet nuclei (yellow, green, and red colors indicate the mucus, protein, and NaCl, respectively).

**FIG. 13.**
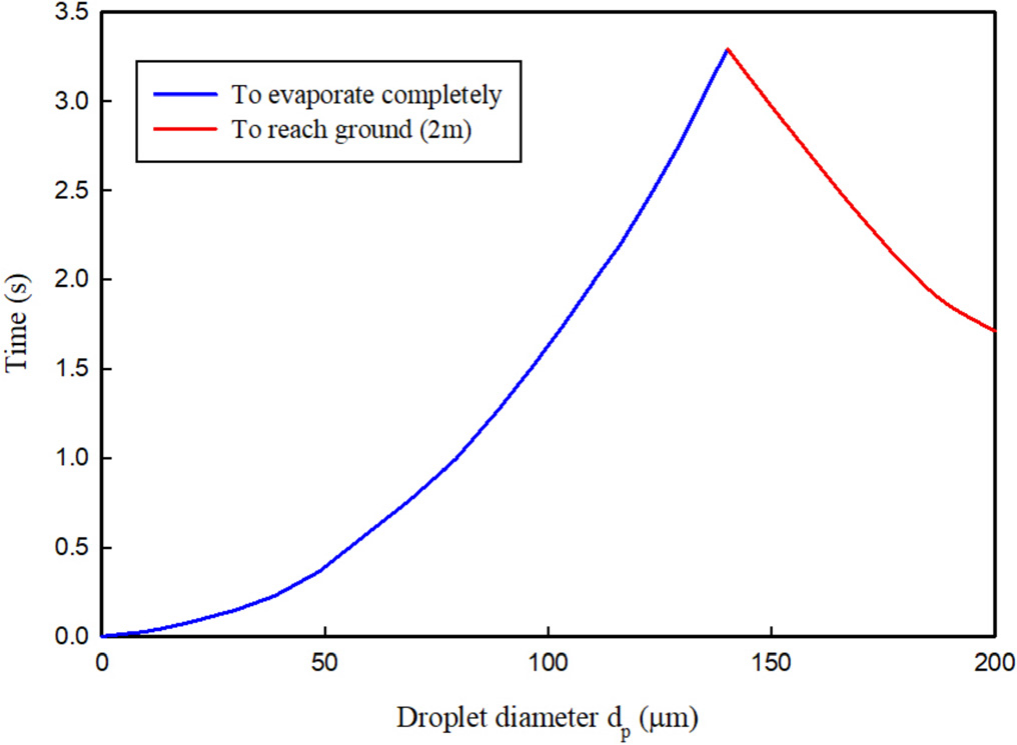
Falling times and evaporation times of droplets.^8^ Reproduced with permission from Wells, Am. J. Epidemiol. **20**, 611 (1934). Copyright 1934 Oxford University Press.

The most fraction of the droplets released by coughing or sneezing is water. These droplets span over a wide size range and about half of the droplets evaporate within several seconds and become droplet nuclei.^62^ In particular, the evaporation process is completed instantaneously for small droplets with an initial size smaller than 20 *μ*m in diameter.^86^ A schematic of the evaporation process and the droplet nuclei formation is shown in Fig. 12. As illustrated after the water evaporates, the droplet nuclei remain, and they are the significant cause of infection in the environment, as first reported by Wells in 1934.^8^ The droplet diameter can play a significant role in the evaporation process. Based on Fig. 13 that is known as Well's curve, the smaller droplets (droplets less than 140 μm) evaporate completely before falling and the droplet nuclei remain suspended in the air. Figure 13, indicates a critical diameter for bio-aerosol particles to deposit on a floor from a 2 m height (i.e., average human height) before dried-out as droplet nuclei. The peak of this curve (intersection point, 140 μm) shows the initial droplet diameter for which the evaporation time is equal to the time for the droplet to reach the ground.

In addition to the effect of droplet diameter on the evaporation process, the composition of droplets released from human respiratory activity also affects the evaporation rate. Drops released from the respiratory activity are different from pure water droplets and contain components such as salt, protein, lipid, carbohydrates, and DNA. Vejerano and Marr^87^ considered the presence of salt (NaCl 9 g/l), protein (mucin 3 g/l), and surfactant (dipalmitoylphosphati-dylcholine, DPPC, 0.5 g/l). DPPC is among the most common phospholipid lung surfactants that decrease surface tension during breathing.^88^

**FIG. 14.**
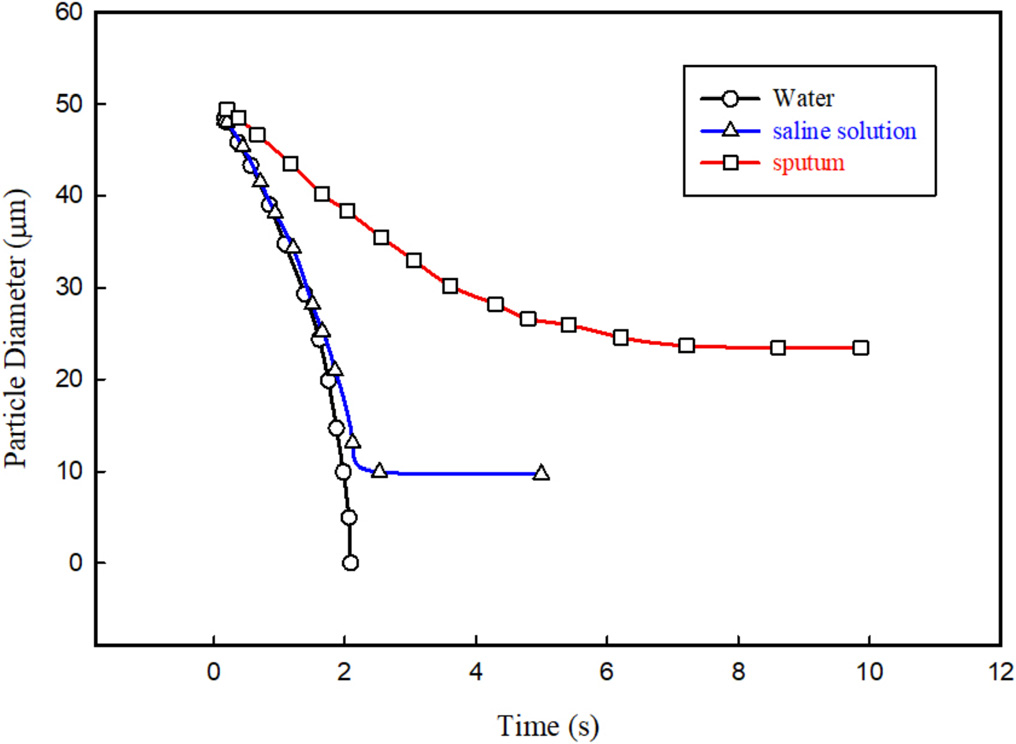
Evaporation of sputum droplets with an initial size of 50 μm from the coughing jet, the relative humidity of ambient air is 80%, and the temperature of ambient air is 294.15 K.^62^ Reproduced with permission from Redrow *et al.*, Build. Environ. **46**, 2042 (2011). Copyright 2011 Elsevier.

The droplet properties (e.g., surface tension, latent heat, and salinity) significantly affect the droplet's evaporation rate. The presence of these components makes the simulation of the droplet evaporation process from human respiratory activities different from the simulation of pure water droplets and makes it more difficult to simulate. Figure 14 shows the evaporation rate of pure water droplets and real cough droplets (obtained from numerical simulations^62^). As can be seen, the sputum droplet constituents have a significant effect on the evaporation time. Based on Fig. 14, a pure water droplet's evaporation rate differs from that of a cough droplet. By presenting this short introduction, we tried to demonstrate the effects of droplet size and sputum droplet constituents on the evaporation rate.

How is the evaporation rate of droplets released from human respiratory activity modeled in the literature? Has the literature paid attention to the thermophysical properties of real respiratory droplets? What relationship was often used to model the heat transfer rate for the droplets? These are the questions that will be addressed in this section.

In recent decades, many experimental investigations have considered pure water droplets and human saliva droplets. Ranz and Marshall examined a distilled water droplet's evaporation rate at a low Reynolds number.^85^ The experiment conditions were: The diameter ranged between 0.6 and 1.1 mm. The experiment was done in still dry air under the environmental temperature of 24.9 °C. The temperature of the droplet was 9.11 °C.

Based on the experimental results,^85^ during 780 s, a water droplet with a diameter of 1 mm evaporates under the mentioned conditions. They confirmed the analogy between heat and mass transfer at a low Reynolds number. Based on Ranz and Marshal,^85^ the fitted mass transfer coefficient and the heat transfer coefficient are as follows:

$$h_{m}=\frac{D_{dv}}{D_{d}}\left(2+0.6{{Re}_{d}}^{0.5}{Sc}^{\frac{1}{3}}\right),$$ (19)

$$h=\frac{D_{dv}}{D_{d}}\left(2+0.6{{Re}_{d}}^{0.5}{Pr}^{\frac{1}{3}}\right),$$ (20)

where ${Re}_{d}=\frac{\left|{\bar{u}}_{i}-{u_{i}}^{p}\right|d_{p}}{\nu }$, $Sc=\frac{\mu }{\rho_{\mathrm{air}}D_{d}}$, and $Pr=\frac{\mu_{\mathrm{air}\;}{C_{p}}_{\mathrm{air}}}{\lambda_{\mathrm{air}}}$ are the particle Reynolds number, the Schmidt number, and the Prandtl number, respectively. Here, ${\bar{u}}_{i}$ is the air velocity, ${u_{i}}^{p}$ is the particle velocity, and ν is the air kinematic viscosity. $D_{d}$ is the diffusivity coefficient of vapor in air, and $\lambda_{\mathrm{air}}$ is the thermal conductivity of the air.

The coefficients were used in mass and heat transfer equations ( $h_{m}\;\mathrm{and}\;h$)^85^

$${\dot{m}}_{e}=\rho_{d}\frac{d}{dt}\left(\frac{\pi }{6}d^{3}\right)=-h_{m}A_{d}(\rho_{v,s}-\rho_{v,\infty }),$$ (21)

$$m_{d}C_{p_{d}}\frac{dT_{d}}{dt}=hA_{d}\left(T-T_{d}\right)+{\dot{m}}_{e}h_{fg},$$ (22)

where ${\dot{m}}_{e}$ is the droplet evaporation rate, $\rho_{d}$ and $A_{d}$ are the droplet density and surface area, respectively, $m_{d}$ is the droplet mass, $C_{p_{d}}$ is the droplet specific heat, and $T_{d}$ and $h_{fg}$ are the droplet temperature and evaporation latent heat, respectively.

In Eq. (22), $\rho_{v,s}=\frac{P_{\mathrm{sat}@T_{d}}}{RT_{d}}$ is the saturation pressure at droplet temperature and $\rho_{v,\infty }=\frac{X_{i}P}{RT}$ is the water vapor density in the surrounding air, where ${\;X}_{i}$ is the local bulk mole fraction of water vapor and R is the universal gas constant.

In the literature, alternative correlations for finding the Nusselt and Sherwood numbers are suggested, e.g., the Abramzon and Sirignano model^89^

$$h=\frac{k\;\ln(1+B_{T})}{D_{d}B_{T}}\left(2+0.6{Re}^{0.5}{Pr}^{\frac{1}{3}}\right),$$ (23)

where k is the thermal conductivity and $B_{T}$ is the Spalding heat transfer number^89^

$$B_{T}=\frac{C_{\mathrm{pv}}\left(T_{a}-T_{d}\right)}{h_{fg}-\frac{q_{d}}{{\dot{m}}_{de}}}\left(2+0.6{Re}^{0.5}{Pr}^{\frac{1}{3}}\right),$$ (24)

where ${\dot{m}}_{e}$ is the droplet evaporation rate and $q_{d}$ is the thermal energy transferred to the droplet. This model is based on a uniform composition and temperature distribution inside the droplet.

Parienta *et al.*, in a mathematical modeling, modified the evaporation rate modeling.^90^ They modeled the droplet evaporation rate of a uniform droplet (the first model) and a droplet with inner composition variation and temperature gradient (the second model). The uniform droplet evaporation rate was calculated based on the Ranz and Marshall correlations,^85^ whereas the droplet with inner composition variation evaporation rate was calculated based on the multiple shells droplet with temperature and concentration gradients. In the second model, the droplet was considered as multiple shells. This model allows accounting for gradients of concentration and temperature within the droplet. In the model, they have assumed:

(1) Each of the shells is treated as uniform concerning temperature and solute concentrations.
(2) The number of shells does not vary during evaporation.
(3) All shells have the same thickness proportional to the diameter of the droplet.
(4) The outer shell evaporates.
(5) There are mass transfer and heat transfer between the shells.
(6) The net mass transfer through each shell is proportional to the mass loss due to evaporation, while the flux of each species is determined by diffusion.

The transient mass and temperature diffusion rates are calculated by^90^

$$\frac{{dm}_{i,x}}{dt}=4\pi {\left(\frac{x-1}{{Sh}_{n}}\right)}^{2}N_{i,x-1}-4\pi {\left(\frac{x}{{Sh}_{n}}R_{d}\right)}^{2}N_{i,x},$$ (25)

where $m_{i,x}$ is the mass of component i in the shell x ranging 1– ${Sh}_{n}$, ${Sh}_{n}$ is the number of shells, $N_{i,x}$ stands for the net mass flux of component i from shell x to shell x + 1, and $R_{d}$ indicates the radius of the droplet.

The net mass flux of component i from shell x to shell x + 1 defined as follows:^90^

$$N_{i,x}=J_{i,x}+\rho_{i,x}\frac{x}{{Sh}_{n}}\left|\frac{{dR}_{d}}{dt}\right|,$$ (26)

where $\rho_{i,x}$ is the concentration (in g/L) of component i in shell x and $J_{i,x}$ is the diffusion flux of component i from shell x to shell x+1.

The diffusion flux $J_{i,x}$ for the solutes was calculated by^90^

Ji,x=−DiRdShnρi,x+1−ρi,x, (27)

where $D_{i}$ is the diffusion coefficient of component i.

The boundary conditions for the net flux are as follows:^90^

$$N_{i,0}=0.$$ (28)

As obtained by symmetry and

$$\begin{array}{c} N_{w,{Sh}_{n}}=K_{d}\left(\rho_{ws}-\rho_{w\infty }\right),\\ N_{sa,{Sh}_{n}}=0,\\ N_{pr,{Sh}_{n}}=0, \end{array}$$ (29)

where $N_{w,{Sh}_{n}}$, $N_{sa,{Sh}_{n}}$, and $N_{pr,{Sh}_{n}}$ are the mass flux of water, salt ions, and glycoproteins on the shell number n ( ${Sh}_{n}$), respectively.

In the modeling, the convective mass transfer coefficients for the mass flux inside the droplet were calculated by the Stokes–Einstein relation and assuming Nu = 2. [Based on Eq. (20) of the convection heat transfer coefficient *h*, the Nusselt number is defined as $Nu=hd/k_{g}$, where d is the droplet diameter and $k_{g}$ is the thermal conductivity of air.] The Stokes–Einstein relation describes spherical molecules so that it can overestimate the diffusion coefficient for the glycoproteins.^90^

They have used the following equations to model the heat transfer between layers and surface droplets:^90^

$$m_{x}C_{l}\frac{{dT}_{x}}{dt}=q_{x-1}4\pi {\left(\frac{x-1}{{Sh}_{n}}\right)}^{2}-q_{x}4\pi {\left(\frac{x}{{Sh}_{n}}R_{d}\right)}^{2},$$ (30)

where $m_{x}$ is the mass of shell x, $C_{l}$ is the heat capacity of water, $T_{x}$ is the temperature of shell x, and $q_{x}$ is the heat flux from shell x to shell x + 1^90^

qx=−kwRdShbTx+1−Tx. (31)

**FIG. 15.**
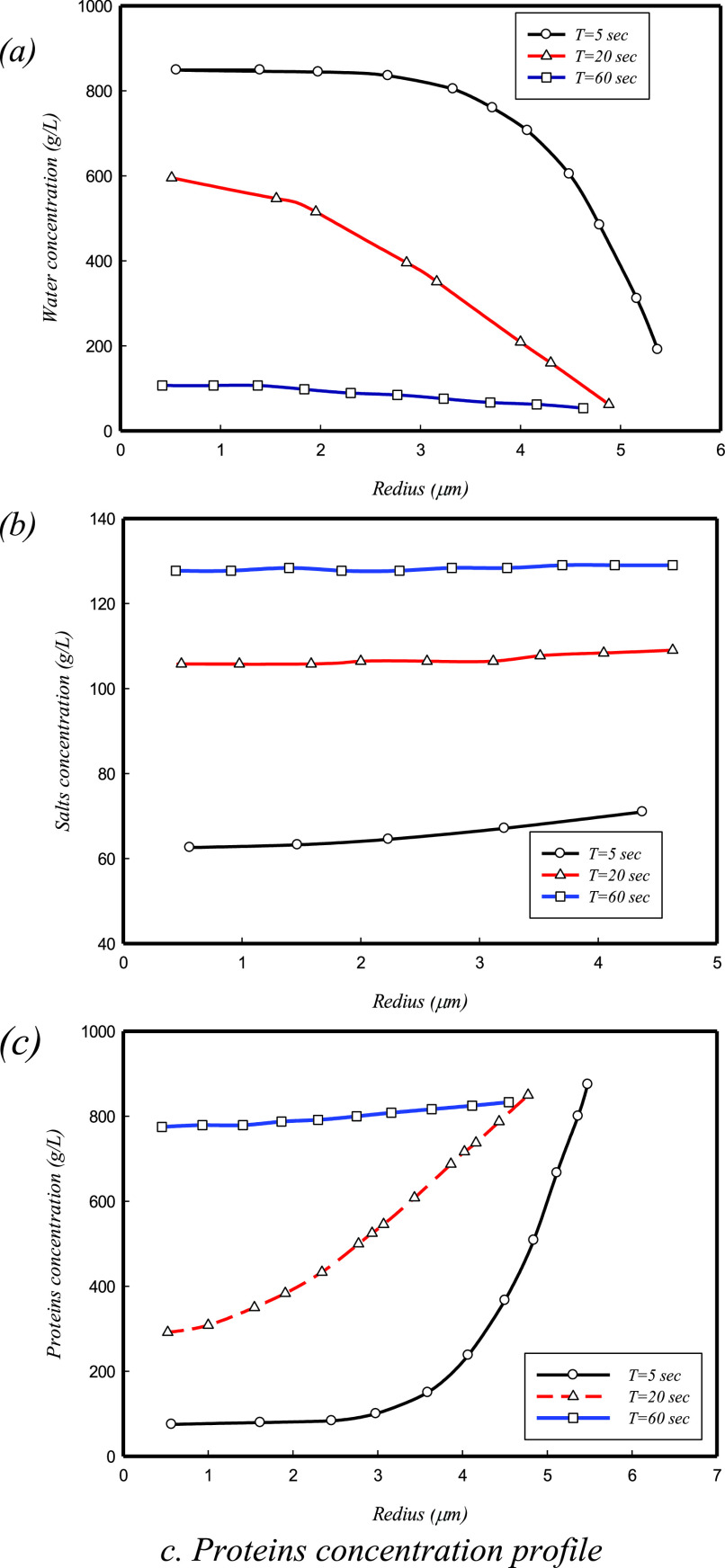
Droplet concentration profile for (a) water, (b) salts, and (c) proteins at several times for a 20 μm droplet.^90^ Reproduced with permission from Parienta *et al.*, J. Aerosol Sci. **42**, 1 (2011). Copyright 2011 Elsevier.

By adapting the following boundary conditions:^90^

$$\begin{array}{c} q_{0}=0,\\ q_{\mathrm{Shn}}=-h\left(T_{\infty }-T_{\mathrm{Shn}}\right)-\rho_{L}L_{t}\frac{dR_{d}}{dt}, \end{array}$$ (32)

where h is the heat transfer coefficient determined by Ranz–Marshall correlations,^85^ $T_{\infty }$ is the temperature of the surrounding air, and $T_{\mathrm{Shn}}$ is the temperature at the surface of the droplet. Based on the results of Parienta *et al.*,^90^ the droplet's evaporation process can be divided into two parts. At the first step, the evaporation rate is similar to pure water, but in the second step, the droplet solute constituents slow down the evaporation rate. In Fig. 15, the droplet concentration profile for water, salts, and proteins is presented in T = 5, 20, and 60 s after droplet injection. In Fig. 15, the horizontal axis is the droplet radius ( μm), and the vertical axis is the component concentration (g/l). As shown, the change in concentrations happens first at the outer shells and moves toward the center of the droplet. Based on Fig. 15(a), over time, the behavior of liquid water concentration is different from the other components. As water evaporates, the concentration of liquid-water droplets is reduced. In contrast, the salt and protein concentrations increase. Based on Fig. 15, if the salt and protein were not modeled, after 60 s, the water concentration would reduce to around 100 g/l. In contrast, salt and protein concentrations increased by more than 120 and 800 g/l, respectively. Also, comparing the salt and protein concentrations [Figs. 15(b) and 15(c)] shows that the profile for salt ions is more moderate than the profile for proteins, because salt ions are smaller than proteins and have a higher diffusion coefficient.

Based on our knowledge and the literature review, in the published numerical investigations, the effect of component concentration on the multi-component respiratory droplet evaporation has not been considered, whereas considering the impact of the composition of the real respiratory droplets can enhance the accuracy of numerical results. High-accuracy numerical results are necessary, because they can help policy setting and governmental actions in the next waves of COVID-19 or pandemic.

Redrow *et al.*^62^ presented a new model to simulate sputum droplets' evaporation and dispersion from human coughs or sneezes. They have simulated the evaporation process of the real sputum droplet containing NaCl, amino acids, ncarbohydrates, and lipids. They used Ranz–Marshall correlations for the single pure water droplet but used the following calculation based on Pruppacher and Klett^91^ to modify it for modeling the evaporation of real cough/sneeze droplets:

$$r\frac{dr}{dt}=\frac{D_{v}M_{w}e_{\mathrm{sat}}\left(T_{a}\right)}{\rho RT_{a}}\{RH-\frac{1}{1+\delta }\exp\left[\frac{L_{v}M_{w}}{RT_{a}}\left(\frac{\delta }{1+\delta }\right)+\frac{2M_{w}\sigma_{s}}{RT_{a}(1+\delta )\rho_{w}r}-\frac{M_{w}\rho_{N}r_{N}^{3}}{\rho_{w}(r^{3}-r_{N}^{3})}\sum_{y}\frac{v_{y}\Phi_{y}\varepsilon_{y}}{M_{y}}\right]\},$$ (33)

where $\frac{D_{v}M_{w}e_{\mathrm{sat}}\left(T_{a}\right)}{\rho RT_{a}}$ is the diffusion term, $\frac{L_{v}M_{w}}{RT_{a}}\left(\frac{\delta }{1+\delta }\right)$ is latent heat term, $\frac{2M_{w}\sigma_{s}}{RT_{a}(1+\delta )\rho_{w}r}$ is surface tension influence, and the last is solute influence, where

$$\delta =\frac{T}{T_{a}}-1=\frac{L_{v}\rho_{s}}{T_{a}k_{a}}r\frac{dr}{dt},$$ (34)

r is the droplet radius, t is the time, $D_{v}$ is the diffusivity of water vapor in air, M is the molecular weight, $T_{a}$ is the local air temperature, T is the instantaneous droplet temperature, $e_{\mathrm{sat}}$ is the saturation vapor pressure over a planar liquid surface at temperature $T_{a}$, ρ is the density, R is the universal gas constant, RH is the relative humidity (as a fraction), $L_{v}$ is the latent heat of vaporization of water, $\sigma_{s}$ is the surface tension, $\Phi_{y}$ is the practical osmotic coefficient, $v_{y}$ is the number of ions into which a solute molecule dissociates, $\varepsilon_{y}$ is the mass fraction of a constituent y with respect to the total dry mass, and $k_{a}$ is the thermal conductivity of continuous phase (air). Details for the calculation ion-interaction parametrization of $\Phi_{y}$ were given in Ref. 92. The subscripts w and s stand for water and solution of droplets, respectively. The subscript N refers to the dry particle, and y refers to a particular constituent of the particle.

The relative humidity (RH) is calculated as follows:

$$RH=\frac{e_{v}}{e_{\mathrm{sat}}},$$ (35)

where the saturation vapor pressure^93^

$$e_{\mathrm{sat}}=6.1121\times \left(1.0007+3.46\times 10^{-6}P\right)\;\text{exp}(\frac{17.502T_{a}}{240.97+T_{a}}),$$ (36)

where P is the ambient air pressure. An expression for the partial vapor pressure $e_{v}$ was developed by^94^

$$e_{v}=\left(\frac{T_{a}-T_{\infty }}{T_{0}-T_{\infty }}\right)e_{v,0}+\left(1-\frac{T_{a}-T_{\infty }}{T_{0}-T_{\infty }}\right)e_{v,\infty }.$$ (37)

In Eq. (38), $T_{0}$ is the initial temperature of the jet expelled from the mouth/nose of a human subject, $T_{a}$ is the local temperature of the air surrounding the droplet, $T_{a}$ is the ambient air temperature (free stream), $e_{v,0}$ is the initial vapor pressure of the jet, and $e_{v,\infty }$ is the vapor pressure of the ambient air.

The rate of multi-component droplet temperature variation considering the effect of surface tension is as follows:^62^

$$\frac{d}{dt}{(T}_{a}-T)=\frac{-3}{r^{2}\rho_{s}c_{ps}}\left\{k_{a}\left(T_{a}-T\right)+L_{v}D_{v}(Q_{\infty }-Q_{\mathrm{surf}})\right\},$$ (38)

where $c_{ps}$ is the specific heat of the droplet at constant pressure, $k_{a}$ is the thermal conductivity of air, and $Q_{\infty }$ and $Q_{\mathrm{surf}}$ are the water vapor density of the ambient air and the water vapor density at the droplet surface, respectively. $Q_{\mathrm{surf}}$ can be calculated as^62^

$$Q_{\mathrm{surf}}=\frac{100M_{w}e_{\mathrm{sat}}}{RT}\exp\left\{\frac{2M_{w}\sigma_{s}}{RT_{a}\rho_{w}r}-\frac{M_{w}\rho_{N}r_{N}^{3}}{\rho_{w}(r^{3}-r_{N}^{3})}\sum_{y}\frac{v_{y}\Phi_{y}\varepsilon_{y}}{M_{y}}\right\}.$$ (39)

More details of the mentioned equations are presented in Ref. 95.

Based on the above discussions, the droplets released from the human respiratory activity have different components that significantly affect the droplet evaporation rate. Therefore, to perform an accurate simulation, it is necessary to model the multi-component droplet. In addition to the evaporating rate of the real respiratory droplets, the virus dose and decay rate of the virus in the droplet is an important factor and should be considered, which is the topic of Sec. VI.

## VI. SURVIVAL OF AEROSOLIZED VIRUS IN THE AMBIENT AIR

In the previous numerical simulations, the droplet trajectories and the traveled distance were predicted without considering the bioactivity of virus-containing aerosols, which makes it difficult to assess the actual effect of virus transport by droplets and aerosols. Our aim of this section is to help provide a more direct indication of the actual virus transport due to droplets and aerosols, by considering the survival rate of the virus. This fills a gap in the current literature, where the numerical simulation of respiratory droplet dispersion does not translate into the risk assessment of virus transport by droplets/aerosols.

In reality, not all of the aerosolized virus remains biologically active, which is a critical factor of viral transmission. The survival ability is virus resistance to physical and biological stresses imposed by the environment.^96^ A virus may not survive from the virology perspective and may not spread the infection along all the dispersion process. In other words, viruses released from respiratory activities have various survival abilities. Virus survival in the ambient air is affected by biological and physical factors.^97^

The most critical aspects affecting virus survival are biological factors like the envelope layer and the virus type. The envelope layer is the outermost layer of many viruses that primarily affect a virus's persistence in the environment. Also, the presence of blood, feces, mucus, and saliva as the surrounding organic material tends one to insulate the virus against extreme environmental changes.^98^

The second factor that affects the decay rate of virus is the physical factors that include the ambient temperature, relative humidity, and sunlight.^99^ Also, the pandemic outbreak models can be affected by the virus decay rate. Recently, Dbouka and Drikakis^100^ presented the vital relationship among weather seasonality, airborne virus transmission, and pandemic outbreaks over a whole year. Based on the Dbouka and Drikakis model,^100^ pandemic outbreaks are linked with changes in temperature, relative humidity, and wind speed independently of the particular season.

Information regarding the environment's influence on all virus decay rates and stability in an external condition is not complete and clear. Depending on the virus type, the effect of the relative humidity varies. Also, the effect of the relative humidity may be different at different temperatures.^101^ Overall, based on the literature, the enveloped viruses, such as influenza and coronaviruses, have higher persistence in lower relative humidity and survived longer.^102^ In contrast, non-enveloped viruses survived longer in higher relative humidity.^103^

**FIG. 16.**
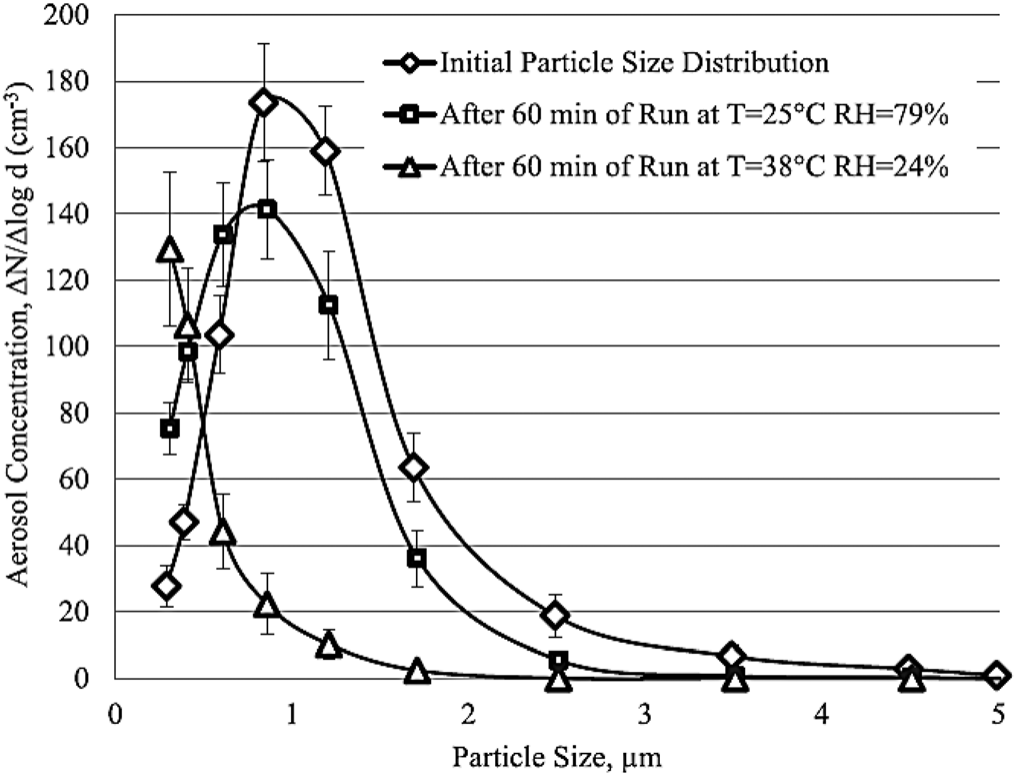
Size distribution of virus-containing particles in the aerosol chamber.^104^ (Error bars represent standard deviation of at least 20 measurements.) Reprinted with permission from Pyankov *et al.*, J. Aerosol Sci. **115**, 158 (2018). Copyright 2018 Author(s), licensed under a Creative Commons Attribution (CC BY) license.

**FIG. 17.**
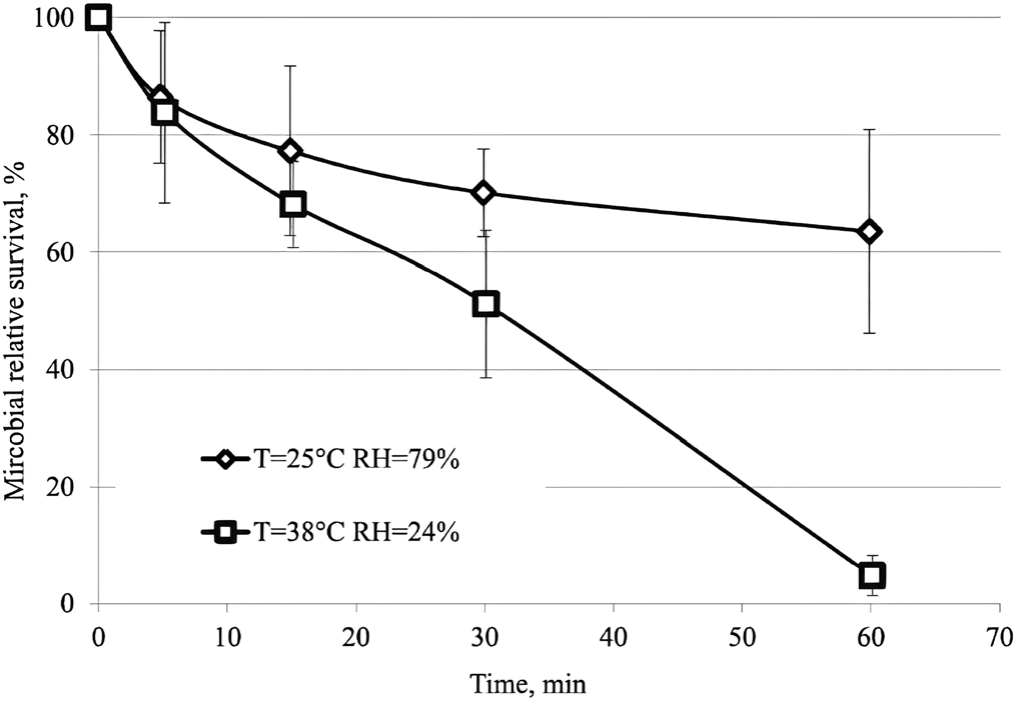
Microbial relative survival in airborne.^104^ Reprinted with permission from Pyankov *et al.*, J. Aerosol Sci. **115**, 158 (2018). Copyright 2018 Author(s), licensed under a Creative Commons Attribution (CC BY) license.

Specifically, we mention the experimental results of Pyankov *et al.*^104^ They investigated the inactivation of aerosolized coronavirus virus in the two ambient air conditions. They tested the survival of influenza viruses after injection into the aerosol chamber. The majority of particles ranged from 0.5 to 2 μm in diameter. They used a rotating aerosol chamber to minimize aerosol settlement under the action of gravitation. They suggested the rotating aerosol chamber for time-related investigations of airborne micro-organism's behavior. In Fig. 16, the initial PSD at the beginning of the test and after 60 min in two different ambient air conditions [air temperature (T = 25 and 38 °C) and relative humidity (RH = 79 and 24%)] is shown. As can be seen, when T = 25 °C and RH = 79%, some shift of the curve obtained after 60 min and droplets' diameter decreases slightly, which indicates minor evaporation of the droplets at these air conditions. Whereas when T = 38 °C and RH = 24%, much more significant changes were seen. As illustrated in Fig. 16, at these conditions after 1 h, the vast majority of particles that remained in the air were smaller than 0.7 μm. In Fig. 17, the microbial relative survival is presented. As seen only over the first 5 min, both curves show a similar trend, and after that, the ambient condition shows its effect on the microbial relative survival. Then their behavior significantly changed, and at the end of the test, when T = 25 °C and RH = 79%, more than 60% virus remain alive whereas at T = 38 °C and RH = 24%, only around 10% of the initial virus remain alive.

**FIG. 18.**
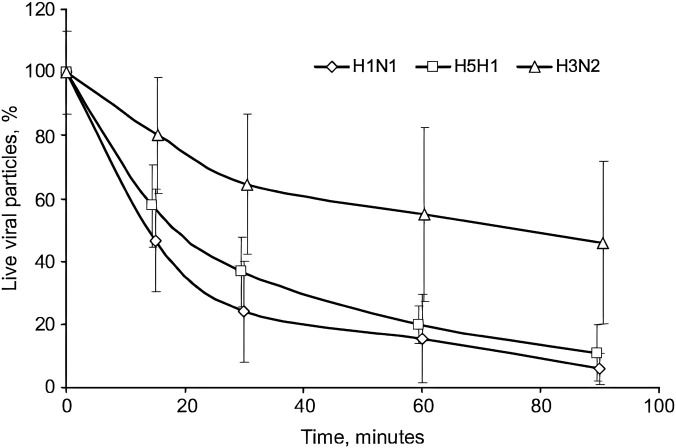
Inactivation of airborne influenza strains of various subtypes over 90 min experimental run.^105^ Reprinted with permission from Pyankov *et al.*, J. Aerosol Sci. **53**, 21 (2012). Copyright 2012 Author(s), licensed under a Creative Commons Attribution (CC BY) license.

As mentioned in Secs. I–V, the PSD of the respiratory droplets was considered in some numerical investigations. The effect of ambient air conditions on the droplet evaporation rate was also modeled in many numerical investigations. However, the decay rate of virus has not been considered and modeled, whereas Pyankov *et al.*^105^ reported different behaviors for three strains of influenza virus under the same ambient air condition and the same initial PSD. They investigated the survival time of three strains of the influenza virus (H1N1, H5N1, and H3N2) in the rotating aerosol chamber. Inactivation of airborne influenza strains of various subtypes over 90 min experimental run is shown in Fig. 18. As can be seen, both H1N1 and H5N1 strains show a close trend, and after 60 minutes, only 20% of the viruses remain alive, while the behavior of the H3N2 virus is different that after 90 minutes, around 50% of them remain alive. These experimental results indicated that the numerical studies without considering the decay rate will have serious uncertainty.

### A. SARS-COV-2 survival

While it remains difficult to provide a general mathematical model for the survival rates of different types of viruses in different environmental conditions, approximate models based on experimental observations would still be much desired. Given the current epidemic around the world, this section mentions some of the studies that examined coronaviruses' survival rate. Only when a survival model is incorporated, numerical simulation results can be connected to risk estimation of the spread of infection in the environment.

Abundant studies have examined how long the coronaviruses remain infectious in aerosols under the different environmental conditions in recent years. Recent studies show that in climate-controlled indoor environments, SARS-CoV-2 SARS (Severe Acute Respiratory Syndrome) - CoV (Corona Virus -2) is relatively stable in aerosols with a decay rate of less than 3% per minute.^96,106,107^ The SARS-CoV-2 decay rate is also affected by the ultraviolet radiation reported in the investigation of Schuit *et al.*^107^ Based on their results, without ultraviolet radiation, every 75 min 90% of infectious virus become inactive whereas with ultraviolet radiation only 8 min is needed to deactivate the same portion of infectious virus.

Dabisch *et al.*^108^ showed for the first time the effect of environmental conditions such as temperature, relative humidity, and simulated sunlight on SARS-CoV-2 decay rate. However, the effects of sunlight and temperature are more significant than humidity. According to their results, more than 2 h was needed for a 90% decrease in infectious viruses without sunlight. They also studied the intensity of simulated sunlight. In the high intensity simulated sunlight (1.9 W/m^2^), virus inactivation took 4.7 min when the temperature and relative humidity were 40 °C and 20%, respectively; whereas 10.9 min was needed when the temperature decreased to 10 °C. Examination of the effect of simulated sunlight intensity has shown that as it increases, the decay rate of virus increases. They have presented a standard regression model for the three factors affecting the decay rate of virus. The complete regression models using relative humidity are presented in Eq. (41)

$$k_{\mathrm{infectivity}}=7.56+1.411\left(\frac{\left(T-20.54\right)}{10.66}\right)+0.022\left(\frac{\left(RH-45.235\right)}{28.665}\right)+7.553\left(\frac{\left(S-50\right)}{50}\right)+1.397\left(\frac{\left(T-20.54\right)}{10.66}\right)\left(\frac{\left(S-50\right)}{50}\right),$$ (40)

where T is the ambient temperature, RH is the relative humidity, and S is the sunlight intensity.

Based on the equation, the simulated sunlight intensity has the greatest influence on the decay rate. Also, the coefficient of relative humidity is much lower than the coefficient of temperature. So, it can be concluded that, the intensity of simulated sunlight and temperature are more important than relative humidity. Based on their results, as the temperature rises, the decay rate of SARS-CoV-2 virus in aerosols has increased, a behavior that has already been reported for other coronaviruses such as 229E and MERS (Middle East Respiratory Syndrome )-CoV in aerosols.^104^ Increasing the decay rate by increasing temperature may modulate the incidence of COVID-19 that has been reported in some statistical studies.^109,110^

**FIG. 19.**
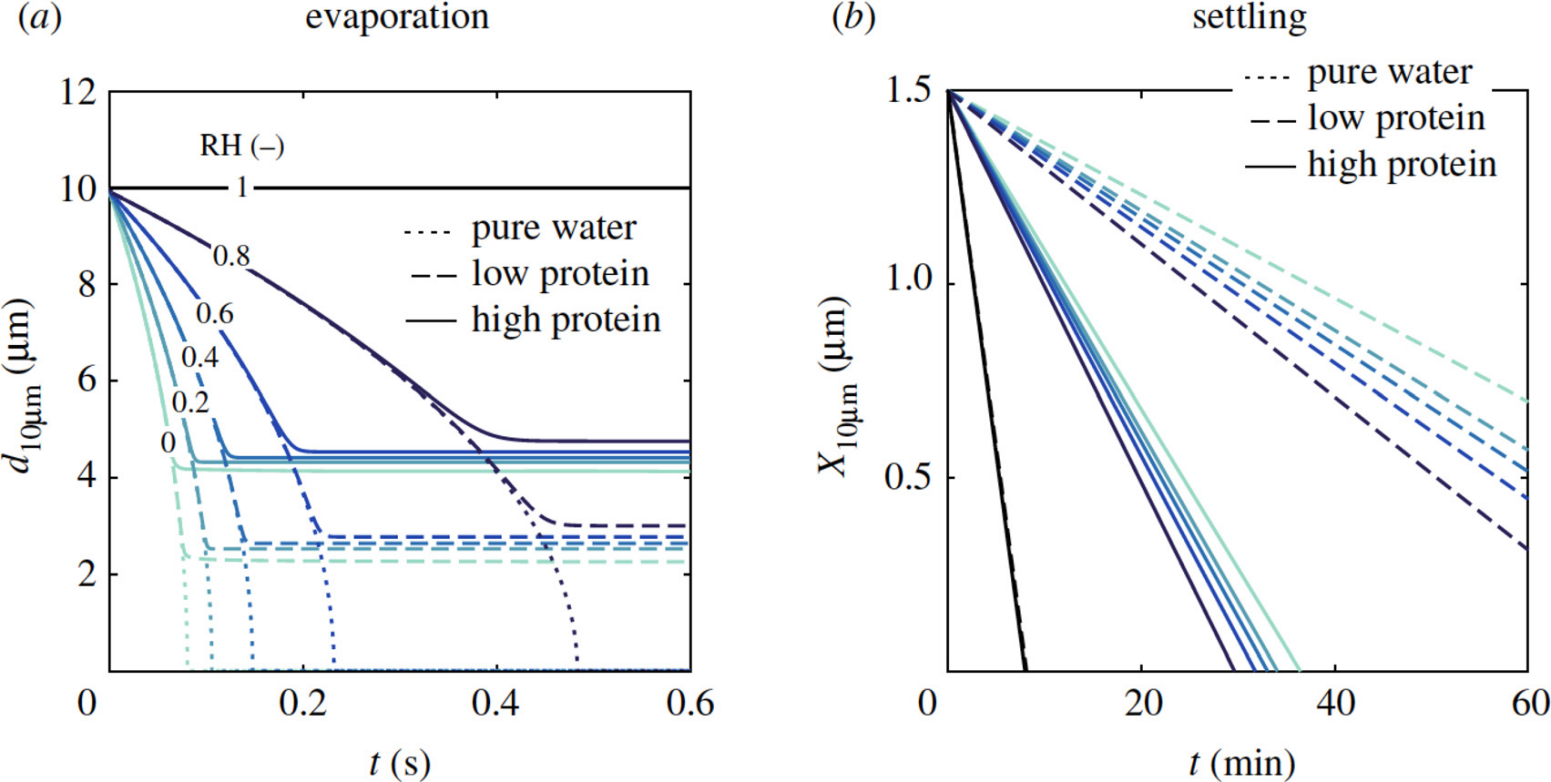
Effect of the droplet composition on (a) the evaporation and (b) the settling of 10 *μ*m droplets under different relative humidity (ambient temperature 20 °C).^111^ Reprinted with permission from de Oliveira *et al.*, Proc. R. Soc. **447**, 20200584 (2021). Copyright 2020 Author(s), licensed under a Creative Commons Attribution (CC BY) license.

Recently, a comprehensive investigation based on the Lagrangian framework has been conducted by de Oliveira *et al.*^111^ They considered the evaporation rate and settling time of the respiratory droplet that released from coughing and speaking. They analyzed the effect of sputum composition of the droplet, temperature, and the relative humidity of the ambient on the evaluated evaporation rate, decay rate, and the total number of suspended viable viral copies for speaking and coughing theatrically. They considered three cases include pure-water, high-protein sputum, and low-protein sputum droplets and modeled the droplet evaporation based on the Abramzon and Sirignano model.^112^ Their novelty is considering the suspended viable virus, decay rate, and infection doses of the droplets. Although their modeling was conducted based on one dimensional theoretical formulation, the mentioned evaluated parameters make their theoretical estimation different from previous studies. The effect of the sputum composition and relative humidity on the evaporation rate and the settling time of 10 μm droplet is presented in Fig. 19. As can be seen, pure water evaporated entirely in less than a second (except when relative humidity is 100%). In contrast, the high and low protein sputum compositions behave differently. As the droplet evaporates, the non-valiant composition concentration increases; thus, the vapor pressure reduces, reaching a larger equilibrium diameter. Therefore, due to the high non-valiant composition concentration in high proteins sputum, the equilibrium diameter of high protein sputum is larger than low protein sputum. As illustrated in Fig. 19(b), the effect of equilibrium diameter on the settling time is significant. As shown, the settling time of the low protein sputum composition is more than high protein sputum composition. Based on Fig. 19(b), the effect of the relative humidity on the evaporation rate is significant, as the evaporation rate increases with decreasing relative humidity. De Oliveira *et al.*^111^ also investigated the virus decay rate in a droplet that assumed an exponential decay such as

$$\frac{{dN}_{k,v}}{dt}=-\lambda N_{k,v},$$ (41)

where $N_{k,v}$ is the total number of viable viral copies in a single droplet k. λ is an exponential decay constant. The value of λ for the SARS-Cov-2 based on the experimental results of Doremalen *et al.*^9^ was considered, namely, $\lambda =0.636\;h^{-1}$. They tested the stability of the SARS-CoV-2 virus formed of 5 μm droplets in ambient air (relative humidity 65%).

**FIG. 20.**
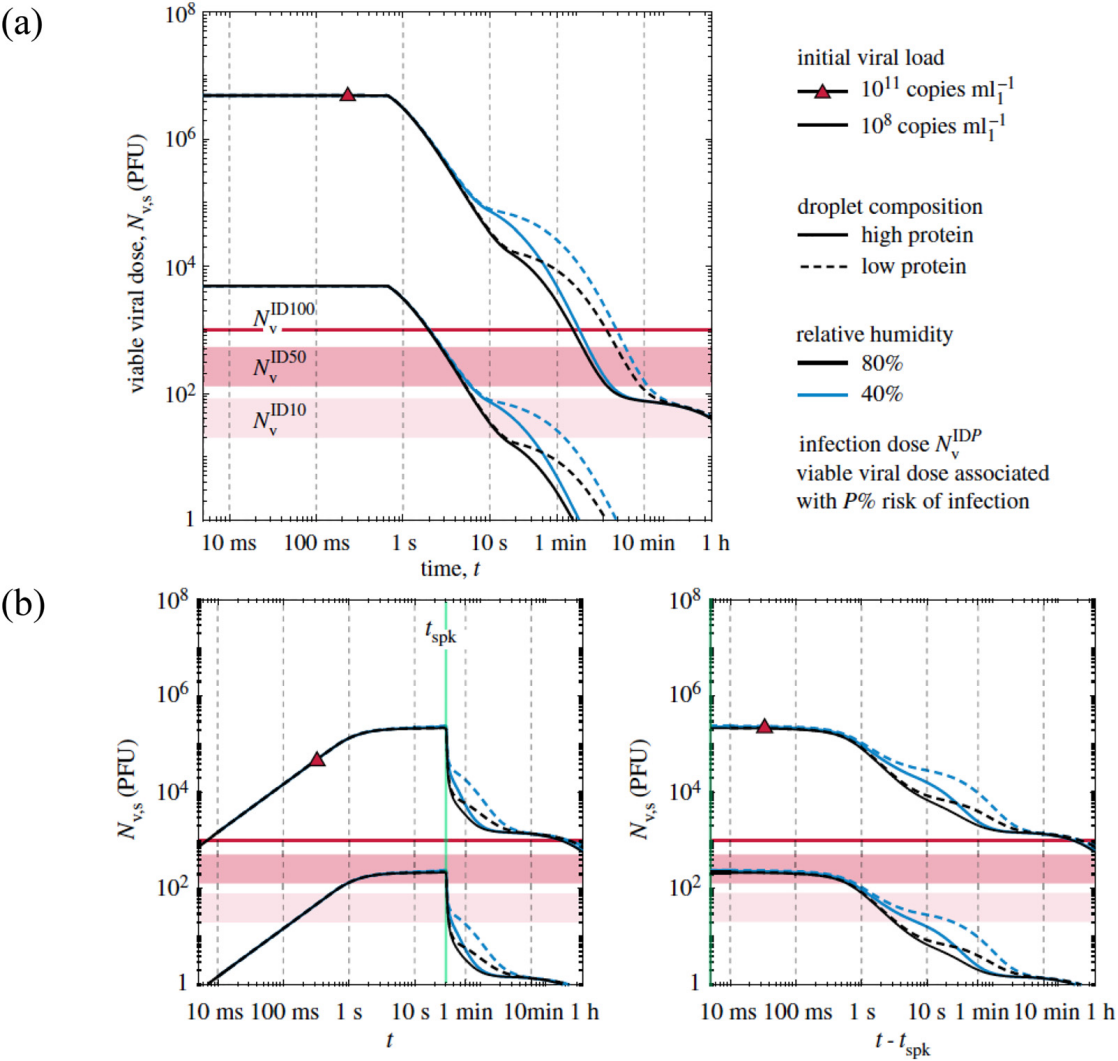
Effect of sputum composition (low- and high-protein sputum), relative humidity (RH = 40% and 80%), and initial viral load $N_{v,0}$ on the suspended viable viral dose during (a) coughing and (b) speaking.^111^ Reprinted with permission from de Oliveira *et al.*, Proc. R. Soc. **447**, 20200584 (2020). Copyright 2020 Author(s), licensed under a Creative Commons Attribution (CC BY) license.

De Oliveira *et al.*^111^ evaluated the suspended viable viral dose in terms of two droplet compositions (low and high-protein sputum), RH = 40% and 80% and low and high initial viral load. Viable viral dose is expressed as $N_{V}^{\mathrm{ID}P}$ and refers to the number of pathogens, including viruses and bacteria, which is sufficient to infect P% of a given susceptible population. One should note that, to date the viable viral dose of SARS-Cov-2 is not available and they used the data from Watanabe *et al.*^113^ Risk of infection P for a viral dose equal to $N_{v}$ is defined as

$$P=1-\exp\left(-\frac{N_{v}}{k_{p}}\right),$$ (42)

where $k_{p}$ is 410 for SARS-CoV-1^113^ and the total suspended viable viral copies $N_{v}$ and viable viral load in copies $V_{v,s}$ are defined as follows:

$$N_{v}=\sum_{k}N_{k,v},$$ (43)

$$N_{v,s}=\frac{N_{v}}{\sum_{k}V_{k}},$$ (44)

where $V_{k}$ is the volume of the droplet. The effect of sputum composition (low- and high-protein sputum), relative humidity (RH = 40% and 80%), and initial viral load $N_{v,0}$ on the suspended viable viral dose is shown in Fig. 20. As can be seen for coughing, in the maximum viral load, about 1–7 min are needed to viral inactivation and bring the total $N_{v,s}$ down to a value below those needed for 100% risk of infection. In opposition to coughing, in speaking 30 min are needed to the same value of $N_{v,s}$. Another important point in Fig. 20 is the effect of relative humidity on the time required for the viral inactivation. As can be seen, with decreasing the relative humidity, the virus inactivation time increases.

In summary, the decay rate and inactivation time of a virus are a complex problem. They depended on many parameters (biological characteristics and ambient air conditions). Incorporation of virus decay into numerical simulation of virus aerosol transport requires cross-disciplinary knowledge transfer between fluid mechanics and virology. Based on the discussion in the current section, there is an important open question: if the COVID-19 virus was non-enveloped virus, would the assumption and results change in the numerical investigation that were published recently? Unfortunately, the answer is no, in the majority studies, because respiratory droplets are considered as pure water in the conducted simulation and the factors that affected the virus decay rate have not been considered. Based on what was reviewed, we think coupling the presented models of decay rate and the suspended viable viral dose in a 3D CFD simulation can eventually allow us to develop a reliable quantitative simulation tool for aerosol virus infection.

## VII. SUMMARY AND CONCLUSIONS

This review addresses critical questions and challenges in the accurate numerical simulation of virus transmission due to human respiratory activities. Although lot of studies have been conducted to address these questions, we point out that many sources of uncertainties remain to be addressed, and how we might handle them.

Specifically, realistic boundary conditions are essential to the accuracy of numerical simulation results. We have discussed some state-of-the-art studies, which provide the necessary information to properly set up the boundary conditions, including the drop size distribution, time-dependent jet inlet velocity profile, etc. Second, droplets from coughing or sneezing have unique composition, mucus and protein, making modeling of their evaporation dynamics particularly challenging. We have provided the state-of-the-art descriptions to help CFD modelers improve their evaporation modeling in the future. Finally, some recommendations are presented to allow researchers to incorporate the decay rate and viable viral dose contained in droplets and airborne virus aerosols, so that their numerical simulations can begin to be used to quantitatively assess the risk associated with the aerosol transmission route in various settings.

By reading this review article, the reader would appreciate and begin to pay attention the following important aspects:

• The duration of coughing and the time-dependent inlet velocity profile significantly affect the penetration depth of coughing.
• More accurate experimental measurements of the coughing droplet size distribution are needed to resolve the reported inconsistencies in the literature and to properly set up future simulations.
• The effect of the composition of real cough droplets on the evaporation rate should be considered in the modeling.
• Two developed and improved models for evaporation rate considering surface tension and the effect of real drop composition are available.
• Preliminary formulation of the virus decay rate and virus dose is also available to use in the future numerical simulations.

Based on the information presented in this review, it is suggested that improved CFD simulations should be conducted to bring CFD results closer to the condition in reality and to better compare with the clinical observation results.

## Data Availability

The data that support the findings of this review are available from the corresponding author upon reasonable request.
